# *Annurca* apple polyphenol extract selectively kills MDA-MB-231 cells through ROS generation, sustained JNK activation and cell growth and survival inhibition

**DOI:** 10.1038/s41598-019-49631-x

**Published:** 2019-09-10

**Authors:** Elisa Martino, Daniela Cristina Vuoso, Stefania D’Angelo, Luigi Mele, Nunzia D’Onofrio, Marina Porcelli, Giovanna Cacciapuoti

**Affiliations:** 1 Department of Precision Medicine, University of Campania “Luigi Vanvitelli”, via Luigi De Crecchio 7, 80138 Naples, Italy; 2 Department of Motor Sciences and Wellness, “Parthenope” University, via Medina 40, 80133 Naples, Italy; 3 Department of Experimental Medicine, University of Campania “Luigi Vanvitelli”, via Luciano Armanni 5, 80138 Naples, Italy

**Keywords:** Apoptosis, Breast cancer

## Abstract

Polyphenols represent the most studied class of nutraceuticals that can be therapeutics for a large spectrum of diseases, including cancer. In this study, we investigated for the first time the antitumor activities of polyphenol extract from *Annurca* apple (APE) in MDA-MB-231 triple negative breast cancer cells, and we explored the underlying mechanisms. APE selectively inhibited MDA-MB-231 cell viability and caused G2/M phase arrest associated with p27 and phospho-cdc25C upregulation and with p21 downregulation. APE promoted reactive oxygen species (ROS) generation in MDA-MB-231 cells while it acted as antioxidant in non-tumorigenic MCF10A cells. We demonstrated that ROS generation represented the primary step of APE antitumor activity as pretreatment with antioxidant N-acetylcysteine (NAC) prevented APE-induced G2/M phase arrest, apoptosis, and autophagy. APE downregulated Dusp-1 and induced a significant increase in JNK/c-Jun phosphorylation that were both prevented by NAC. Moreover, downregulation of JNK by its specific inhibitor SP600125 significantly diminished the anticancer activity of APE indicating that ROS generation and sustained JNK activation represented the main underlying mechanism of APE-induced cell death. APE also inhibited AKT activation and downregulated several oncoproteins, such as NF-kB, c-myc, and β-catenin. In light of these results, APE may be an attractive candidate for drug development against triple negative breast cancer.

## Introduction

Breast cancer represents the principal cause of cancer death among women in developed countries^1^. The triple-negative breast cancer (TNBC; estrogen receptor-negative, progesterone receptor-negative and HER2-negative)^2^ is often characterized by high level of mutated p53 and is considered as highly aggressive form of cancer with poor disease-free and overall survival^3^. Currently, TNBC treatment is mainly through chemotherapy, to which it is highly resistant^3^. Therefore, the identification of new targeted therapies against TNBC represents an important clinical challenge.

Adverse toxic side effects of chemotherapy during breast cancer treatment have shifted considerable focus towards anti-cancer natural compounds as a valuable source for new drug development. Indeed, dietary components and natural products enhance the efficacy of standard chemotherapy by overcoming drug resistance and by reducing toxicity and side-effects^4,5^.

Among natural compounds, polyphenols have been widely studied for their bioactive properties and for their therapeutic roles in the prevention of cardiovascular and neurodegenerative diseases and cancer^6^. Most of the dietary polyphenols display cancer preventive and antitumor activities both in animal models and in humans. Polyphenols are good candidates to be used as inhibitors of tumor cells growth^7^ for their potential to regulate the activity of several gene targets involved in carcinogenesis, through both direct interaction and modulation of their expression. Notably, a peculiar feature of polyphenols as chemotherapeutic drugs is their ability to efficiently kill tumor cells while affecting only marginally normal cells^8^.

Although most of the beneficial effects of natural polyphenols can be ascribed to their antioxidant properties as scavengers of free radical generated from various sources in the environment as well as from cellular processes^9^, emerging evidences indicate that polyphenols may also have pro-oxidant activity and modulate chemical signaling pathways which definitively lead to antiproliferative and apoptotic effects in pre-neoplastic or neoplastic cells^6^. ROS play a major role in carcinogenesis^10^ and contribute to the antitumor activity of several chemotherapeutic drugs. However, pro-oxidant molecules could be potential cytotoxic agents by increasing ROS levels of cancer cells beyond critical threshold limits^10^. Cancer cells, being under improved oxidative stress, are more sensitive to ROS than normal cells^11^. Therefore, polyphenol-induced ROS production could be an effective strategy for the selective killing of cancer cells^11^.

Accumulating evidences indicate that excessive ROS can induce cell death by modulating c-Jun-N-terminal kinase (JNK), a stress-associated protein kinase belonging to mitogen-activated protein kinase (MAPK) family^12^. JNK is susceptible to ROS response and is dragged into various cellular processes, including autophagy and apoptosis^13^. Hence, targeting the ROS/JNK signaling pathway might be effective for the treatment of TNBC^14^.

Apple fruits are one of the most important sources of polyphenolic compounds in the Western diet^15^. Apple polyphenols have shown high antioxidant capacity *in vitro*^15,16^ and it has been described that the consumption of apple increases the antioxidant status of blood^16^, exhibits an anti-arteriosclerosis activity by reducing low-density lipoprotein oxidation^17^, and decreases cellular glucose levels and lipid uptake^18^. Furthermore, it has been reported that apple polyphenols inhibit the proliferation of cancer cells^15^ and that phenolic phytochemicals present in the apple skin are able to prevent tumor formation in different types of cancer, including breast^19^. *Malus pumila Miller* cv. *Annurca*, an apple variety with a “Protected Geographical Indication” of the Campania region^20^ which accounts for 5% of italian apple production, is extremely rich in catechin, epicatechin, and chlorogenic acid and is characterized by a stronger antioxidant activity than other varieties^21^. Previous results from our laboratory have described the antiproliferative effect of polyphenol extract from *Annurca* apple (APE) in human HaCaT keratinocytes and in human breast carcinoma MCF-7 cells^22,23^.

In the current study, we reported the anticancer effect of APE on triple negative MDA-MB-231 human breast carcinoma cells and we explored the underlying molecular mechanism. We provided evidence that APE induced cell cycle arrest, intrinsic and extrinsic apoptosis, and beclin-independent autophagic cell death through ROS generation, sustained JNK/c-Jun signaling activation and inhibition of survival and growth pathways. We also demonstrated that APE selectively acted as toxic pro-oxidant agent on MDA-MB-231 cells while it displayed a protective antioxidant effect on MCF10A, a non-tumorigenic human mammary epithelial cell line. To our knowledge, this is the first study investigating the antitumor activity of APE in TNBC.

## Results

### APE selectively inhibited the viability of MDA-MB-231 triple-negative breast cancer cells

**Figure 1:**
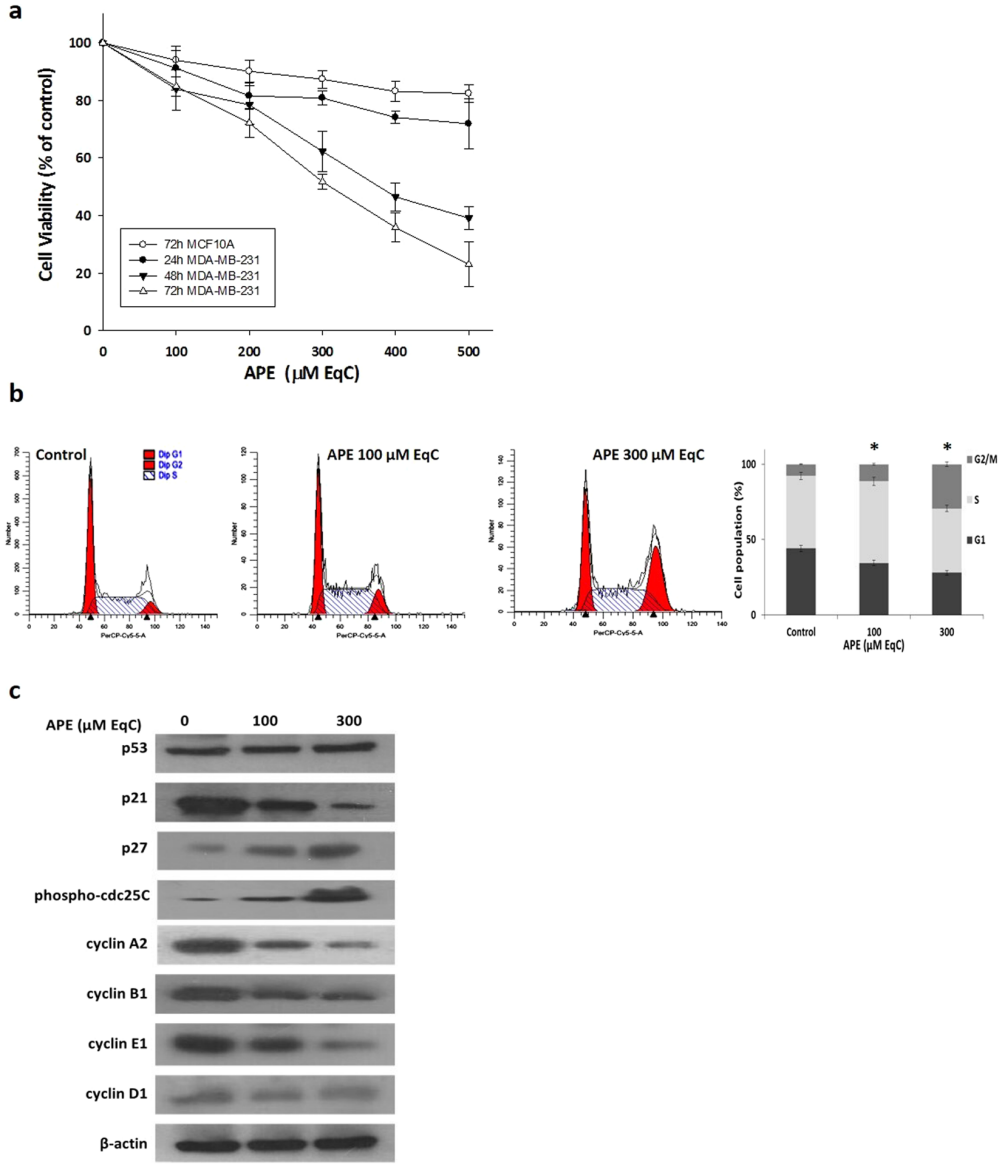
APE inhibits MDA-MB-231 cell growth and induces G2/M phase arrest. (**a**) Effect of APE on MDA-MB-231 and MCF10A cell viability. MDA-MB-231 and MCF10A cells were cultured for 24, 48, and 72 h in medium supplemented or not (control) with APE 100, 200, 300, 400, and 500 μM EqC. Cell viability was then assessed by MTT assay and expressed as a percentage of untreated cells. Values represent the mean ± SD of three independent experiments. (**b**) MDA-MB-231 cells were treated with APE 100 and 300 μM EqC for 24 h. The distribution of cell cycle was assessed by flow cytometry. PI fluorescence was collected as FL3-A (linear scale) by the ModFIT software (Becton Dickinson). For each sample at least 2 × 10^4^ events were analyzed in at least three different experiments giving a SD less than 5% (*P < 0.05 *versus* control). (**c**) The levels of cell cycle-regulatory proteins in MDA-MB-231 cells treated with APE 100 and 300 μM EqC for 24 h were measured by western blotting. β-actin was used as a standard for the equal loading of protein in the lanes. The full-length blots are included in the supplementary information (Fig. S1).

To evaluate the antitumor activity of APE, we first tested its effect on cell viability of MDA-MB-231 and MCF10A cells. When cells were treated with increasing APE concentrations from 100 to 500 μM catechin equivalent (EqC), 29–145 μg EqC/ml, for different times a statistically significant time- and dose-dependent inhibition of growth occurred. The effect was evaluated by MTT assay and resulted in IC_50_ values of 378 and 308 μM EqC at 48 and 72 h, respectively. In contrast, MCF10A cells were affected only minimally since about 85% cell viability was still observable after 72 h at 500 μM EqC APE concentration (Fig. 1a) suggesting that APE specifically targeted cancer cells.

### APE induced G2/M cell cycle arrest through a p53/p21-independent pathway

To identify the underlying mechanism of APE-mediated growth inhibition, we analyzed by flow cytometry cell cycle progression in MDA-MB-231 cells treated for 24 h with APE 100 and 300 μM EqC. As shown in Fig. 1b, APE induced a remarkable dose-dependent accumulation of cells in G2/M phase. Indeed, the G2/M population increased significantly from 7.7% in control to 11.3% and 29.41% in treated cells. To elucidate the mechanism of APE-induced cell cycle arrest at G2/M phase the protein levels of several key cell cycle regulators were examined by western blotting. Fig. 1c shows a notable dose-dependent decrease of cyclin A2, B1 and E1 compared to untreated cells while no significant differences were detected for cyclin D1. We then examined the levels of phospho-cdc25C and cyclin-dependent inhibitors p27 and p21. Results showed a substantial dose-dependent increase of p27 and phospho-cdc25C levels. Interestingly, p21 content was significantly decreased while p53 remained almost unchanged suggesting that APE could retard MDA-MB-231 cell proliferation *via* cyclin downregulation and p27and phospho-cdc25C upregulation to arrest cell cycle progression at the G2/M phase. The observed unmodified p53 level together with the remarkable downregulation of p21 suggested that APE-induced cell cycle arrest is p53/p21-independent.

### APE induced apoptosis through the intrinsic and extrinsic pathway

**Figure 2:**
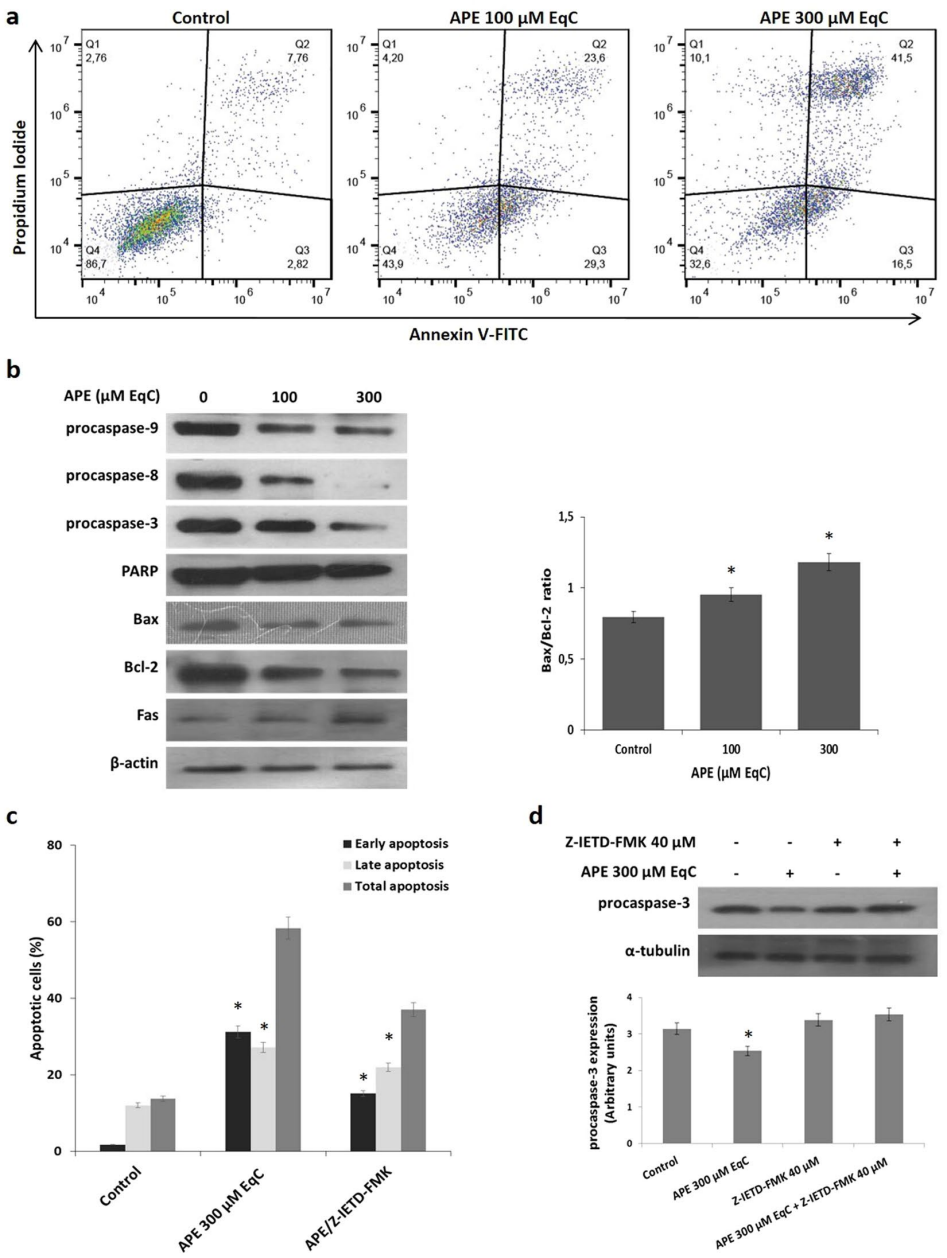
APE induces apoptosis in MDA-MB-231 cells. (**a**) MDA-MB-231 cells were treated with APE 100 and 300 μM EqC for 24 h. Flow cytometry analysis was then performed. Representative dot plots of both Annexin V-FITC and PI-stained cells. The percentage of cells is reported in the quadrants. Viable cells, lower left; non-viable necrotic cells, upper left; early apoptotic cells, lower right; late apoptotic cells, upper right. For each sample 2 × 10^4^ events were acquired. Analysis was carried out by triplicate determination on at least three separate experiments. (**b**) MDA-MB-231 cells were incubated with APE 100 and 300 μM EqC for 24 h. Cell lysates were prepared and analyzed by western blotting assay for the expression of apoptosis-related proteins. The graph shows the densitometric intensity of Bax/Bcl-2 ratio. The intensities of signals were expressed as arbitrary units. The house-keeping protein β-actin was used as loading control. (**c**) MDA-MB-231 cells were treated with APE 300 μM EqC for 24 h with or without co-treatment with 40 µM Z-IETD-FMK caspase-8 inhibitor and cell apoptosis was evaluated by flow cytometry. (**d**) The level of procaspase-3 in MDA-MB-231 cells treated with APE 300 μM EqC for 24 h with or without Z-IETD-FMK was measured by western blotting. The graph shows the densitometric intensity of caspase 3 band under the different experimental conditions. All results were obtained from at least three independent experiments (*P < 0.05 *versus* control).The full-length blots are included in the supplementary information (Figs S2, S3).

In order to investigate whether the growth inhibition upon APE treatment was associated with induction of apoptotic cell death, MDA-MB-231 cells were treated with APE 100 and 300 μM EqC and the apoptotic process was evaluated after 24 h by flow cytometry. As showed in Fig. 2a, APE induced a significant dose-dependent increase of apoptotic cells that reached 58% of the control with APE 300 μM EqC. The results also evidenced a concentration-dependent accumulation of late apoptotic cells that increased from 23,6% to 41,5% suggesting significant dose-dependent morphological changes correlated with the apoptotic process.

To explore the mechanism of apoptosis-mediated cell death, we next analyzed the levels of cleaved caspases and PARP. Fig. 2b shows that APE induced a remarkable concentration-dependent decrease of procaspase 3 and procaspase 9, the initiator caspase of the mitochondrial pathway. Activation of procaspase 3 was further evidenced from the cleavage of PARP. These results indicated that APE promoted apoptosis *via* a caspase-dependent mechanism. To confirm the induction of intrinsic apoptotic pathway, we evaluated the levels of Bax and Bcl-2 proteins, two mitochondria-associated modulators of apoptosis. The balance of these pro- and anti-apoptotic members of Bcl-2 gene family has been thought to determine the functional integrity of the mitochondrial outer membrane and the commitment to apoptotic cell death in mammalian cells^24^. It has to be noted that Bcl-2 serves as the mitochondrial gate-keeper and is frequently overexpressed in tumors where it plays a critical role in chemo- and radio-resistance^24^. Findings indicated that the content of Bax slightly decreased after APE treatment while Bcl-2 levels were intensely reduced. The consistent downregulation of Bcl-2, even in the presence of slightly decreased, albeit consistent, Bax levels, resulted in an increased Bax/Bcl-2 ratio of about 1.5-fold compared to untreated cells (Fig. 2b), indicating that APE-induced apoptosis in MDA-MB-231 cells might occur through the mitochondrial pathway. Fig. 2b also shows that caspase 8, a key protein in the extrinsic apoptotic pathway, was significantly activated by APE. This prompted us to examine whether death receptor Fas played any role in our model. We found that Fas levels increased in a dose-dependent manner following APE treatment indicating that APE induced apoptosis in MDA-MB-231 cells through FAS-mediated pathway. Pretreatment of cells with the specific caspase-8 inhibitor Z-IETD-FMK consistently reduced APE-induced accumulation of apoptotic cell population (Fig. 2c) and reverted procaspase-3 cleavage (Fig. 2d) indicating that the activation of caspase 8 by FAS-mediated signaling played an important role in APE-induced apoptotic cell death. All the data above indicated that APE could efficiently induce apoptosis in MDA-MB-231 triple negative breast cancer cells by activating both the extrinsic and intrinsic apoptotic pathways.

### APE induced beclin-1-independent autophagic cell death in MDA-MB-231 cells

**Figure 3:**
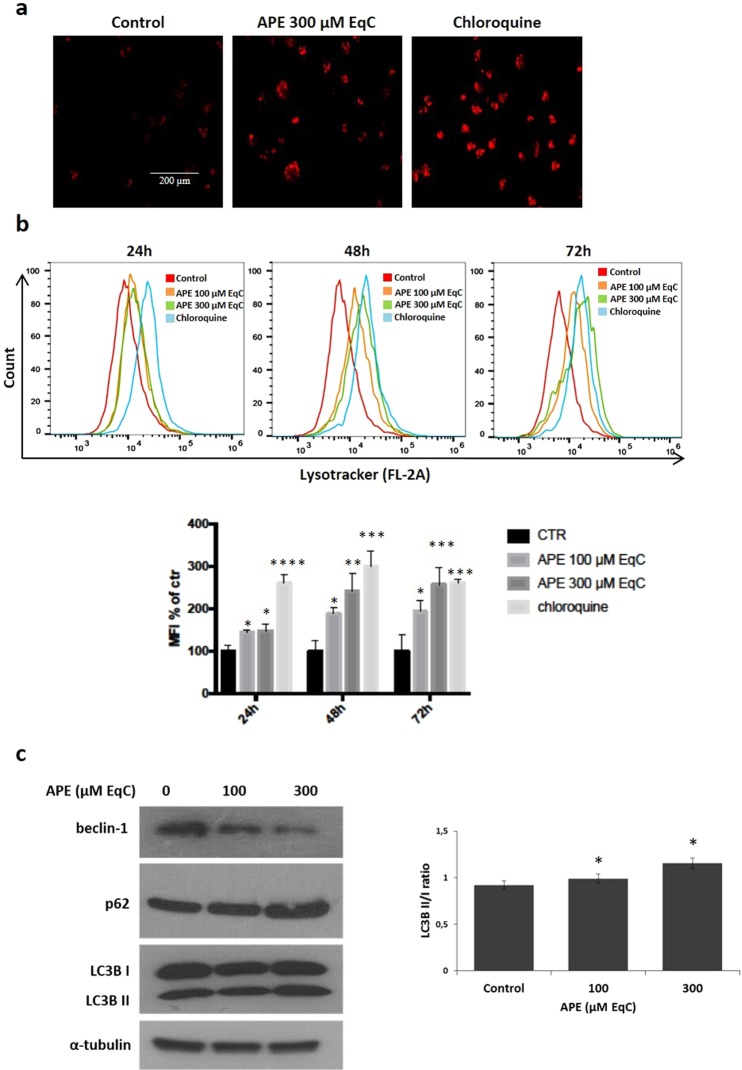
APE induces beclin-independent autophagy in MDA-MB-231 cells. (**a**) Representative images of LysoTracker Red staining of MDA-MB-231 cells treated or not (control) with APE 300 μM EqC for 24 h and analyzed by fluorescence microscopy. Chloroquine is used as positive control. (**b**) MDA-MB-231 cells were treated with APE 100 and 300 μM EqC at indicated times and then flow cytometry analysis was performed. At least 2 × 10^4^ events were acquired in log mode. For the quantitative evaluation of LTR, FlowJo software was used to calculate median fluorescence intensities (MFI) by the formula (MFI-treated/MFI-control). The hystogram represents the values of MFI of LTR analyzed by flow cytometry, as a percentage of the control (*P < 0.05, **P < 0.01, ***P < 0.001, ****P < 0.0001 *versus* control cells). Analysis was carried out by triplicate determination on at least three separate experiments. (**c**) MDA-MB-231 cells were treated with APE 100 and 300 μM EqC for 48 h. The protein content of LC3B, p62, and beclin-1 was analyzed by western blotting. The graph shows the densitometric intensity of LC3B II/I bands ratio. The intensities of signals were expressed as arbitrary units (*P < 0.05 *versus* control cells). α-tubulin was used as loading control. The full-length blots are included in the supplementary information (Fig. S4).

Persistent autophagy in response to oxidative stress serves as a potent death signal^25^. To examine whether APE was able to induce autophagic cell death in MDA-MB-231 cells, we analyzed autophagosome formation after staining with LysoTracker Red (LTR), an acidotropic dye for staining and tracking cellular acidic compartments including autophagosome and autolysosome structures in living cells^26^. As shown in Fig. 3a, the treatment of MDA-MB-231 cells with 300 μM EqC APE caused after 24 hours an increased formation of red dotted acidic vacuoles in comparison with control cells. Next, the quantitative analysis by flow cytometry of the autophagic flux in cells treated for 24, 48 and 72 h with 100 and 300 μM EqC APE evidenced a notable increase respect to untreated cells, indicating the induction of a dose- and time-dependent autophagy (Fig. 3b). To verify the above findings, the protein levels of some markers of autophagy were analyzed by western blotting after 48 h APE-treatment. A characteristic feature of the autophagic process is the conversion of LC3B from LC3BI (cytosolic form) into a lipidized LC3BII (autophagosome membrane-bound) form. As shown in Fig. 3c, APE induced a concentration-dependent accumulation of LC3BII protein. We also evaluated the level of p62/SQSTM1 (p62), a widely used autophagy marker. We found that APE induced a dose-dependent upregulation of p62, in agreement with reports showing that an increase in p62 expression is required for autophagy induction^27^. We then evaluated the protein levels of beclin-1, an important autophagic player involved in the autophagosome formation^28^, that resulted significantly decreased highlighting that APE induced-autophagy in MDA-MB-231 cell is a beclin-1-independent process.

### APE selectively promoted ROS generation in MDA-MB-231 cells while acting as antioxidant in MCF10A cells

**Figure 4:**
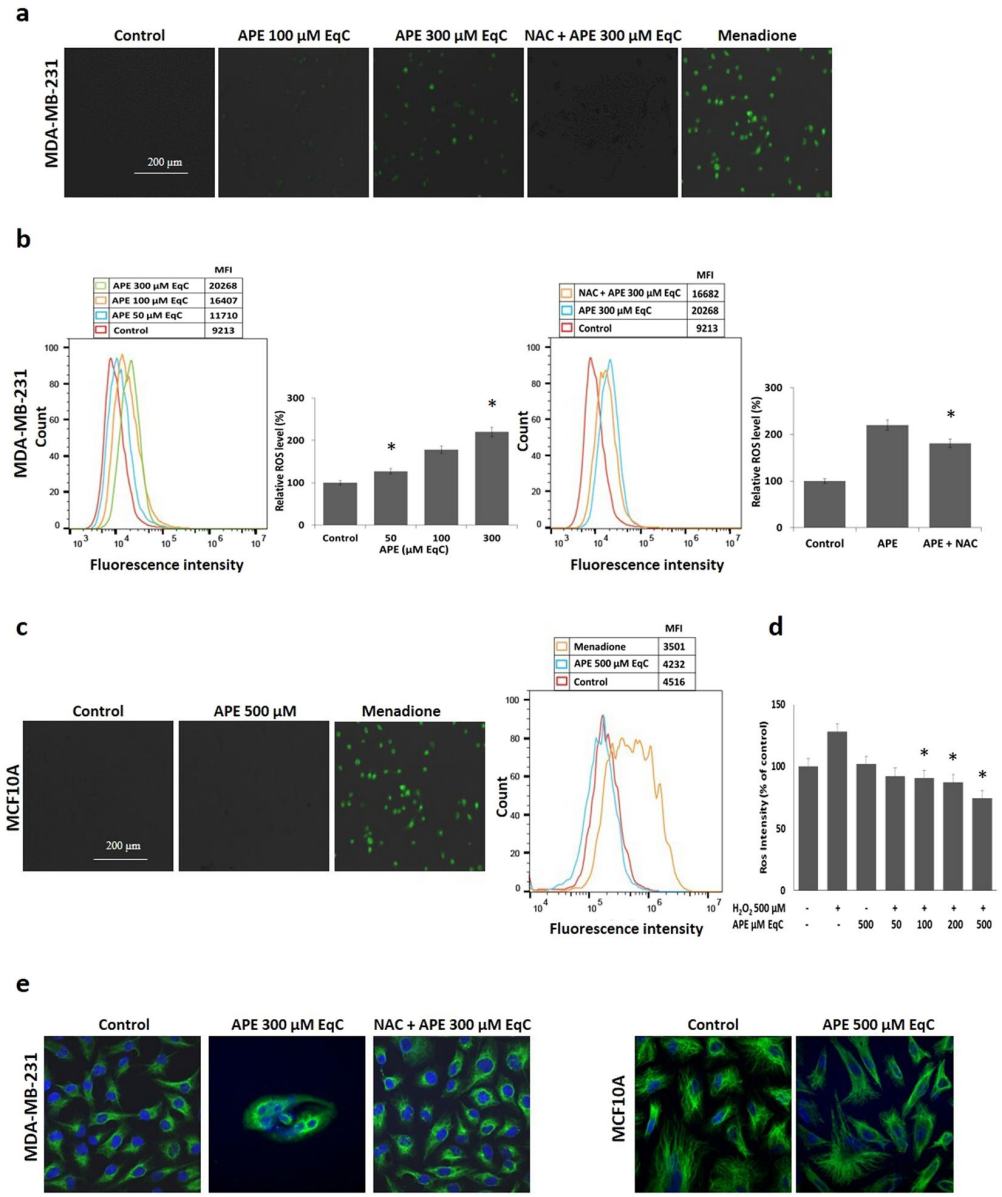
APE increases ROS accumulation in MDA-MB-231 cells, while it displays an antioxidant effect in MCF10A cells. (**a**) Representative images of CellROX staining for oxidative stress determination analyzed by fluorescence microscopy 24 h post-treatment of MDA-MB-231 cells with 100 and 300 µM EqC APE alone or in combination with NAC 5 mM. Menadione is used as positive control while NAC is used as ROS scavenger. (**b**) MDA-MB-231 cells were treated for 24 h with 50, 100, and 300 µM EqC APE alone or in combination with NAC 5 mM and then subjected to flow cytometry to measure ROS levels. MFI values are reported. (**c**) MCF10A cells were treated or not (control) with 500 µM EqC APE for 24 h and, after CellROX staining, analyzed by fluorescence microscopy and FACS analysis. Menadione is used as positive control, MFI values are reported. (**d**) Inhibitory effect of APE on H_2_O_2_-induced oxidative stress in MCF10A cells. Cells were incubated for 24 h with or without APE at the indicated concentrations prior to H_2_O_2_ 500 µM exposure for 30 min. ROS were detected with DCFH-DA and the fluorescence intensity was calculated. Data were expressed as a percentage of untreated control. *P < 0.05 *versus *H_2_O_2_ 500 µM treated cells. (**e**) Representative confocal images of the effect of APE on cell and nuclear morphology in MDA-MB-231 and MCF10A cells treated or not (control) with 300 and 500 µM EqC APE, respectively, alone or in combination with NAC 5 mM for 24 h. Nuclear DNA was labelled with DAPI (shown in blue) while vimentin antibody (in green) was used as cytoskeleton marker. The image of APE-treated MDA-MB-231 cells showing DAPI staining, vimentin staining, and the merge is included in the supplementary information (Fig. S5).

Overproduction of ROS caused damage to the cells and was involved in the regulation of a variety of cellular processes including autophagy, apoptosis, and cell cycle arrest^29^. Therefore, we decided to use the fluorescent CellROX kit staining to monitor intracellular ROS production in MDA-MB-231 and in MCF10A cells by fluorescence microscope and flow cytometry. Exposure of MDA-MB-231 cells to APE 100 and 300 μM EqC resulted, after 24 h, in a substantial dose-dependent increase in the fluorescent CellROX signal as compared with the control (Fig. 4a). This effect was reverted by pretreatment with the antioxidant N-acetylcysteine (NAC) confirming APE-induced ROS generation. Next, we analyzed ROS production by flow cytometry (Fig. 4b). Treatment for 24 h with APE from 50 to 300 μM EqC increased the proportion of cells with elevated green fluorescence confirming the accumulation of intracellular ROS in MDA-MB-231 cells. NAC treatment inhibited the fluorescent signal induced by menadione, thus confirming that the signal was specifically produced by ROS increase. While in MDA-MB-231 cells APE caused a concentration-dependent increase of ROS up to 2.2-fold relative to untreated control cells (Fig. 4b), in MCF10A cells no ROS elevation was observed (Fig. 4c), even at 500 μM EqC APE concentration, suggesting that the induction of ROS was specific for cancer cells. Results also showed that MDA-MB-231 cells were characterized by a basal level of ROS twice higher than that of MCF10A cells confirming the view that cancer cells show persistently high levels of ROS compared to normal cells^11,30^.

To investigate whether APE could act as antioxidant in non-tumorigenic MCF10A cells, we analyzed its effect against H_2_O_2_-induced oxidative stress. The results shown in Fig. 4d demonstrated that pretreatment with increasing concentrations of APE from 50 to 500 μM EqC decreased the level of H_2_O_2_-induced ROS in a dose-dependent way providing evidence of the dual pro-oxidant/antioxidant activity of APE in tumor and in normal cells, respectively.

The morphology of cells is closely related to cellular physiological status and functions. In order to investigate if ROS production might be involved in APE-induced morphological changes, MDA-MB-231 and MCF10A cells were treated with APE 300 and 500 μM EqC, respectively, for 24 h and cell morphology was then analyzed with a confocal microscope. Compared with the corresponding untreated cells, APE-treated MDA-MB-231 cells displayed significant morphological changes, characteristic of apoptotic cells, such as loss of cell adhesion, membrane shrinkage, destruction and collapse of the cytoskeleton and the expulsion of the cell nucleus, cell rounding, condensed chromatin and nuclear fragmentation, and reduced cell density (Fig. 4e). Pretreatment with NAC restored the initial cell morphology confirming that ROS are important mediators of the cytotoxic effect exerted by APE in MDA-MB-231 cells. As expected, no appreciable APE-induced morphological changes were observed in MCF10A cells indicating the absence of APE-induced oxidative stress in non-tumorigenic cells.

### APE induced ROS-mediated JNK activation and Dusp-1 downregulation

**Figure 5:**
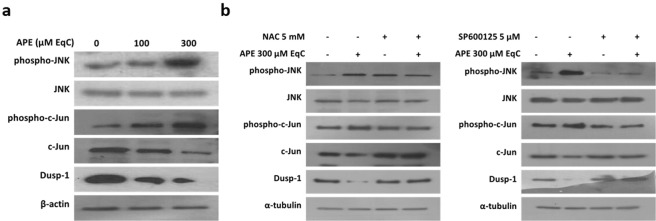
APE-induced ROS activate JNK/c-Jun signaling and downregulate Dusp-1 in MDA-MB-231 cells. (**a**) MDA-MB-231 cells were incubated with APE 100 and 300 μM EqC for 24 h. Cell lysates were prepared and analyzed by western blotting for the expression of phospho-JNK, JNK, Dusp-1, phospho-c-Jun and c-Jun. (**b**) Cells were treated with 300 μM EqC APE for 24 h with or without 1 h pretreatment with 5 mM NAC or 5 µM SP600125. Levels of phospho-JNK, JNK, Dusp-1, phospho-c-Jun and c-Jun were analyzed by western blotting. All results were obtained from at least three independent experiments. The full-length blots are included in the supplementary information (Figs S6, S7).

c-Jun N-terminal kinase is a crucial player downstream of ROS in the molecular pathways leading to cell death. Therefore, we examined if APE-induced ROS were involved in the activation of JNK/c-Jun pathway. The results showed that treatment of MDA-MB-231 cells with APE 100 and 300 μM EqC for 24 h increased the level of phosphorylated JNK and c-Jun in a concentration-dependent manner (Fig. 5a) and that pretreatment with NAC reverted JNK/c-Jun phosphorylation (Fig. 5b) indicating that APE-induced JNK/c-Jun activation is mediated by ROS.

Dusp-1 is a dual-specificity phosphatase that selectively dephosphorylates MAPKs and is the main phosphatase that inactivates JNK^31^. As a result of Dusp-1 increase, JNK activation would be inhibited, which consequently protects tumor cells from JNK‐induced apoptosis^31^. Based on these considerations, we examined Dusp-1 protein level in MDA-MB-231 cells in response to APE. Western blotting analysis evidenced that APE remarkably decreased the level of Dusp-1 in concentration-dependent manner (Fig. 5a) and that NAC completely abolished the effect of APE (Fig. 5b), strongly suggesting that ROS are important mediators of APE-induced Dusp-1 downregulation. Interestingly, Dusp-1 downregulation was also reverted by SP600125 furnishing evidence that JNK is able to modulate Dusp-1 expression.

### APE induced cell cycle arrest, apoptosis, and autophagy through the activation of ROS/JNK pathway

**Figure 6:**
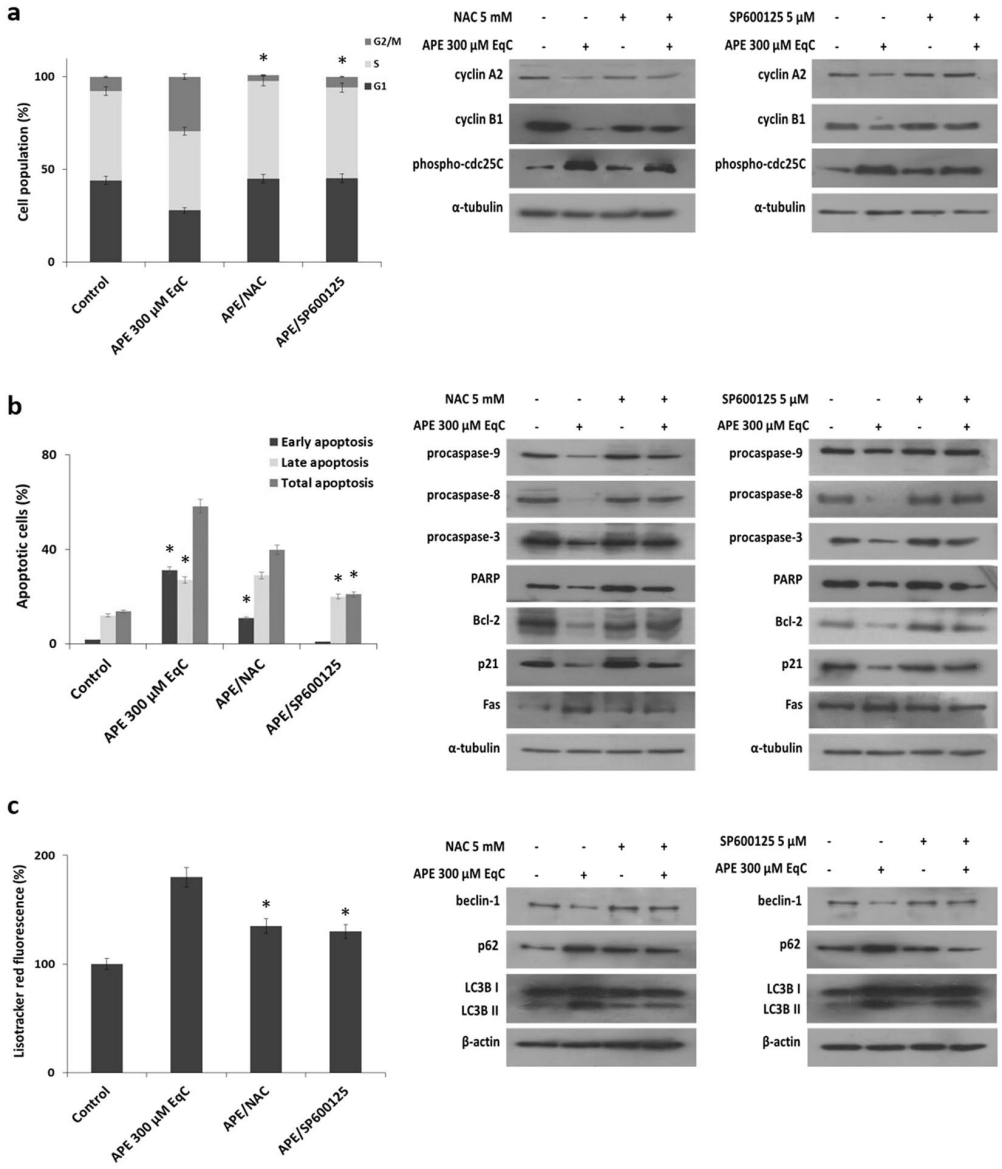
ROS/JNK pathway mediates APE-induced cell cycle arrest, apoptosis, and autophagy in MDA-MB-231 cells. MDA-MB-231 cells were treated with APE 300 μM EqC with or without pretreatment with 5 mM NAC or 5 µM SP600125 for 1 h. (**a**) After 24 h the distribution of cell cycle was assessed by flow cytometry and the expression of cell cycle-related proteins was measured by western blotting. (**b**) After 24 h cell apoptosis was evaluated by flow cytometry and the expression of apoptosis-related proteins was measured by western blotting. (**c**) After 48 h autophagy was analyzed by flow cytometry and the levels of beclin-1, LC3B and p62 were analyzed by western blotting. All results were obtained from at least three independent experiments (*P < 0.05 *versus* control cells). The full-length blots are included in the supplementary information (Figs S8–S11).

We examined whether APE-induced cell cycle arrest, apoptosis, and autophagy could be mediated by ROS generation and JNK activation. To investigate about cell cycle arrest and apoptosis MDA-MB-231 cells were pretreated 1 h with NAC and SP600125 before treatment with APE 300 μM EqC for additional 24 h. Flow cytometric analysis revealed that both molecules almost completely reverted APE-induced G2/M phase arrest and restored the levels of relevant G2/M phase proteins (Fig. 6a). We further found that pretreatments with NAC and SP600125 consistently impaired the APE effect on apoptosis, as detected either by Annexin V/PI staining and western blotting (Fig. 6b). These results indicated that APE induced G2/M cell cycle arrest and apoptosis through ROS-dependent activation of JNK signaling. Next, we investigated the involvement of ROS/JNK axis in APE-induced autophagy. Also in this case either NAC and SP600125 deeply reverted the autophagic process, suppressed the increased levels of LC3BII and p62, and restored the levels of beclin-1 (Fig. 6c). Altogether these results indicated that the activation of ROS/JNK signaling represents the mechanism underlying APE-induced G2/M phase arrest, apoptosis, and autophagy in MDA-MB-231 cells.

### APE inhibited AKT activation and downregulated NF-kB, c-myc, and β-catenin signaling

**Figure 7:**
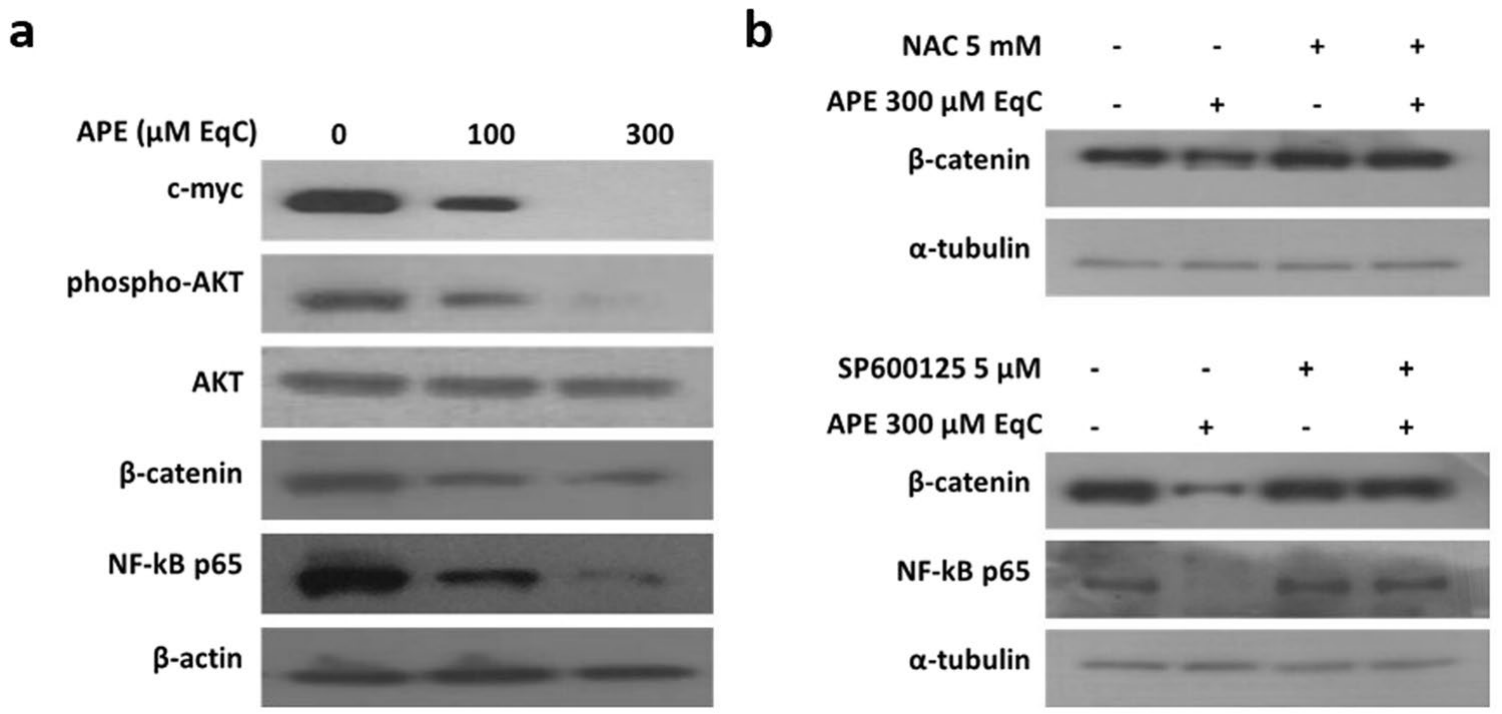
APE downregulates the main cell growth and survival pathways. MDA-MB-231 cells were treated with APE 100 and 300 μM EqC for 24 h with or without pretreatment with 5 mM NAC or 5 µM SP600125 for 1 h. The protein levels of phospho-AKT, AKT, β-catenin, NF-kB p65 and c-myc were measured by western blotting by using the total cell lysate. All results were obtained from at least three independent experiments. The full-length blots are included in the supplementary information (Figs S12, S13).

To further investigate the mechanism of anticancer activity of APE in MDA-MB-231 cells, several cancer-related signaling pathways were analyzed by western blotting after treatment of cells with APE 100 and 300 μM EqC for 24 h. As shown in Fig. 7, APE induced a significant dose-dependent downregulation of c-myc, NF-kB, and β-catenin and strongly inhibited AKT phosphorylation. Interestingly, pretreatment with NAC and SP600125 restored β-catenin level and SP600125 was able to revert NF-kB downregulation. On the contrary, neither NAC nor SP600125 abolished the decrease of c-myc and phospho-Akt suggesting that APE-induced downmodulation of these pathways is independent from ROS/JNK signaling.

## Discussion

New advances in anticancer drug discovery using natural compounds have been made in the last years and polyphenols have emerged as promising molecules being able to act as selective cytotoxic agents leading to tumor cell death through generation of toxic levels of ROS. The present study showed that APE exhibited anticancer activities in MDA-MB-231 triple negative breast cancer cells and provided experimental evidences on the underlying mechanisms. We found that APE selectively and efficiently causes a time- and dose-dependent inhibition of MDA-MB-231 cell viability by a pro-oxidant cytotoxic effect while in non-tumorigenic MCF10A cells it exerted an antioxidant protective effect against the H_2_O_2_-induced oxidative stress. These results are in agreement with recent reports indicating that natural products such as, isoliensinine^32^, physagulide P^33^ and Ziyuglycoside II^34^ are able to selectively increase ROS levels in MDA-MB-231 cells thus allowing to take APE into consideration as a promising novel natural agent for the selective targeting of these TNBC cells.

We provided direct evidence that ROS generation represented the primary step of APE antitumor activity in MDA-MB-231 cells. In fact, pretreatment with ROS scavenger NAC prevented APE-induced inhibition of cell proliferation, as well as G2/M cell cycle arrest, apoptosis, and autophagy.

JNK plays a critical role in natural compounds-induced apoptosis suggesting a tumor-suppressive role of JNK signaling in cancer^13,35^. Consistent with these findings we found that APE induced a notable increase of JNK/c-Jun phosphorylation that is prevented by NAC. Moreover, the inhibition of JNK/c-Jun activation by SP600125 significantly diminished the anticancer activity of APE indicating that the increase of ROS generation and the subsequent activation of JNK cascade play a crucial role in growth inhibition and in programmed cell death induction in MDA-MB-231 cells in response to APE.

The duration of JNK activation is thought to be a critical factor in determining cell proliferation or apoptosis, with transient activation driving essentially prosurviving responses and prolonged activation promoting cell death by mechanisms that are cell type- and stimuli-dependent^13,36,37^. ROS are able to maintain a prolonged JNK activation by inhibiting Dusp-1 through reversible oxidation of a catalytic-site cysteine^38,39^. We found that APE-induced cytotoxicity in MDA-MB-231 cells was associated with a significant increase of JNK/c-Jun phosphorylation and with a concomitant decrease of Dusp-1 level. Moreover, ROS scavenger NAC could efficiently attenuate the effect of APE on either JNK and Dusp-1 indicating that ROS triggered both processes and confirming that in MDA-MB-231 cells Dusp-1 downregulation underlied a mechanism of sustained JNK activation. Interestingly, we found that also JNK, once activated, was able to downregulate Dusp-1 thus providing a positive feedback loop that contributed to potentiate its activation.

Many anti-cancer agents reduce malignant growth by arresting cell cycle at G1/S or G2/M phase^40^. Therefore, cell cycle arrest, particularly at G2/M, might be a useful therapy to prevent the proliferation of cancer cells. Cdc25 phosphatases regulate cell cycle progression in S phase and mitosis by removing inhibitory phosphate groups from cyclin-dependent kinases^41^. G2/M checkpoint is largely mediated through phosphorylation of cdc25C that downregulates the phosphatase activity. It has to be noted that this cell cycle regulatory mechanism is p53-independent and could therefore be particularly relevant for the DNA damage response in cancer cells characterized by high levels of mutant p53, such as MDA-MB-231 cells^36,38^. Interestingly, APE-induced G2/M phase arrest in MDA-MB-231 is accompanied by a notable increase of phosho-cdc25C protein. SP600125 almost completely abolished the effect of APE on cell cycle and significantly reduced phosho-cdc25C levels indicating that, in agreement with literature^41^, JNK-dependent phosphorylation of cdc25C in MDA-MB-231 cells could function as a metabolic checkpoint for the G2/M phase transition. APE-induced G2/M arrest and cdc25C phosphorylation were also reverted by NAC highlighting a link between oxidative stress signals and cell cycle control.

APE simultaneously triggered both extrinsic and intrinsic apoptosis. NAC and SP600125 were able to revert the effects of APE on either apoptosis and on downregulation of all examined apoptosis-related markers, indicating that intrinsic and extrinsic apoptotic pathways are modulated by ROS/JNK signaling. Although autophagy acts primarily as a pro-survival mechanism during nutrient starvation, under some situations it can stimulate a non-apoptotic form of programmed cell death. We found that APE induced autophagy in MDA-MB-231 cells and that pretreatment with NAC and SP600125 reverted the process demonstrating the involvement of ROS/JNK signaling. Strikingly we found that APE-induced autophagy is beclin-1-independent. This evidence as well as the finding that autophagy inhibition attenuated cell growth inhibition strongly suggested that APE-induced autophagy could function as a pro-death mechanism in MDA-MB-213 cells. This hypothesis is supported by several considerations: (i) beclin-independent autophagy is invariably associated with cell death^27,42^; (ii) autophagic cell death is considered a major non-apoptotic cell death mechanism regulated by JNK^43,44^; (iii) in cancer cells, under certain conditions, a sustained JNK activation is crucial to induce autophagic cell death^43,44^; (iiii) during apoptosis, caspase-mediated cleavage of beclin-1 generates fragments that translocate to the mitochondria and induce apoptosis, resulting in an amplified apoptotic cell death^45^. Notably, we found that NAC and SP600125 were able to revert the APE-induced decrease of beclin-1 levels indicating that beclin-1 degradation is dependent from ROS/JNK signaling and strongly suggesting that it could serve as a mechanism to enhance the apoptotic response of MDA-MB-231 cells to APE.

In addition to acting as a powerful cell death inducer, APE also displayed potent inhibitory effects on the expression and activity of several oncoproteins related to cell survival and proliferation. We provided evidence that APE-induced downmodulation of AKT, NF-kB and β-catenin could represent a strategy to enhance a sustained activation of JNK.

First, APE notably downregulated the levels of p21, the cyclin-dependent kinase inhibitor, indicating that G2/M arrest in MDA-MB-231 cells is a p21-independent process. In recent years evidence has been accumulated showing that p21 behaves like a two-faced regulator. Indeed, depending on cell type, cellular localization, p53 status, and the type and level of genotoxic stress, p21 can acquire either oncosuppressive or oncopromoting properties^46^. Regulation of breast cancer greatly depends on the subcellular localization of p21. Nuclear p21, indeed, has a tumor-suppressor activity inducing temporary arrest of cell cycle or leading to a chronic state of senescence or apoptosis. On the other hand, cytosolic p21 displays oncogenic activities by downregulating apoptotic response and promoting cell proliferation^47–49^. The phosphorylation of p21 by AKT is crucial to localize p21 at cytosolic level where p21 can directly bind to JNK and function as a non-competitive inhibitor preventing the activation of JNK and inhibiting its catalytic activity^50^. We demonstrated that APE inhibited AKT activation and induced a significant decrease of p21 levels that was rescued by SP600125 indicating that JNK is responsible for p21 downregulation. Interestingly, recent reports revealed that p21 downregulation *via* ER stress/JNK/caspase-3 axis is required for the antiproliferative activity of the ER stress activator 3-AWA^51^ and that the inhibition of AKT by 3-AWA is a key factor to modulate p21 expression in ER stress conditions^51^. On the basis of these experimental evidences we could hypothesize that APE-induced AKT inhibition could be needed to prevent the cytosolic localization of p21 and its inhibitory effect on JNK allowing JNK to induce p21 downregulation through an analogous ROS/JNK/caspase axis in order to sustain its own activation.

Second, NF-kB is one of the major chemoresistance-related anti-apoptotic factors. High NF-kB activity has been identified in drug-resistant cancer cell lines^52^. Moreover, the anti-cancer activities of many anti-inflammatory drugs are, at least in part, related to the inhibition of NF-kB^53^. NF-kB and JNK signaling pathways are functionally interconnected and the anti-apoptotic function of NF-kB is mediated in part by its ability to downregulate JNK activation^54^. ROS act as mediators between the NF-kB-induced cell survival and JNK-induced cell death. ROS, indeed, are strong activators of NF-kB which in turn inhibits the accumulation of ROS through the upregulation of antioxidant proteins thus hampering ROS-dependent JNK activation and its pro-apoptotic effects^52^. Interestingly, we found that APE downregulated

NF-kB thus providing an additional mechanism to sustain JNK activation. The ability of SP600125 to revert APE-induced downregulation of NF-kB establishes a positive feed-back loop that might further sustain JNK activation.

Owing to the cross-talk between NF-kB and ROS/JNK axis, the ability of APE to simultaneously activate ROS/JNK proapoptotic pathway and inhibit ROS/NF-kB anti-apoptotic pathway might provide a powerful new adjuvant for breast cancer treatment.

Third, recent studies indicate that Wnt/β-catenin signaling is particularly over-activated in TNBC cells where it is involved in several tumor-associated properties, such as migration, stemness, anchorage-independent growth, and chemosensitivity^55^. We demonstrated that APE caused a decrease of β-catenin level in MDA-MB-231 cells that is prevented by NAC and SP600125, thus providing evidence that oxidative stress and JNK negatively regulates β-catenin signaling. Our data are also in agreement with previous reports indicating that in HEK293T cells the activation of JNK1 inhibits Wnt/β-catenin signaling^56^ and that in epidermal cells ROS-induced apoptosis is accompanied by caspase-dependent degradation of β-catenin^57^. Based on these findings, it is possible to hypothesize that APE-induced downregulation of β-catenin could take place through ROS/JNK-mediated caspase activation and could contribute to maintaining a sustained JNK activation in turn responsible for the inhibition of the tumorigenic activities related to β-catenin signaling.

**Figure 8:**
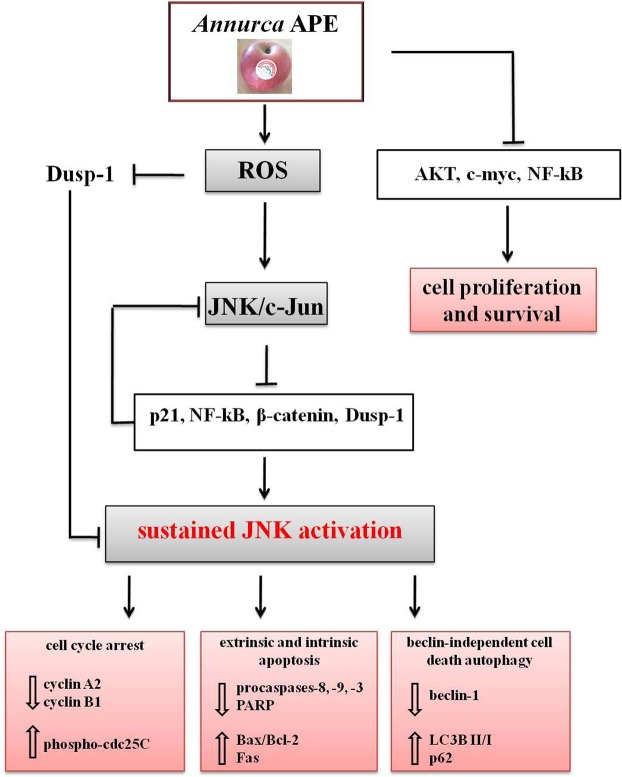
Schematic diagram summarizing the anticancer activities of *Annurca* apple polyphenol extract (APE) in MDA-MB-231 cells and the proposed underlying mechanism.

Figure 8 summarized the multi-targeted effects exerted by APE in MDA-MB-231 cells. In this scenario ROS are the initial players and JNK, like a capable conductor, orchestrates and modulates numerous cellular events that ultimately resulted in cell death. The timing and duration of JNK activation determine whether cells proliferate or undergo programmed cell death. Consistent with this view, we furnished evidences strongly suggesting that the sustained JNK activation, induced by the pro-oxidant effect of APE, could represent the key mechanism to trigger cell death in the highly resistant and invasive MDA-MB-231 breast cancer cells.

In conclusion, our study for the first time highlighted APE as a multi-faceted agent able to modulate the growth of triple negative MDA-MB-231 breast cancer cells through the simultaneous regulation of multiple cell signaling pathways involved either in cell death as well as in cell proliferation and survival. The ability of APE to affect cancer cells at multiple levels and, thus, to potentially circumvent the development of resistance, together with its capability to selectively kill tumor cells while exerting a protective antioxidant effect in normal cells, stimulate great interest in future investigations and make APE an attractive candidate for drug development against TNBC.

## Methods

### Chemicals and antibodies

Propidium iodide (PI), N-acetyl-L-cysteine (NAC), 3-(4,5-dimethylthiazol-2-yl)-2,5-diphenyltetrazolium bromide (MTT), Folin-Ciocalteu reagent, 2′,7′-dichlorofluorescin diacetate (DCFH-DA), chloroquine, 4′,6-diamidino-2-phenylindole (DAPI) nucleic acid stain and the monoclonal anti-vimentin antibody were purchased from Sigma-Aldrich (St. Louis, MO). LysoTracker Red DND-99 (LTR) was obtained from ThermoFisher Scientific (Waltham, MA). Annexin V-fluorescein isothiocyanate (V-FITC) Apoptosis Detection kit and Z-IETD-FMK caspase-8 inhibitor were provided from BD Biosciences (San José, USA). SP600125 JNK inhibitor was obtained from Selleck Chemicals (Munich, Germany). Monoclonal antibodies to caspases 8 and 9, poly(ADP ribose) polymerase (PARP), Bax, Bcl-2, p21, p53, beclin-1, β-catenin, AKT, phospho-AKT, Fas, cyclin E1, cyclin A2, cyclin D1, c-myc, NF-kB p65, phospho-c-Jun, c-Jun, β-actin, α-tubulin and polyclonal antibodies to caspase 3, phospho-cdc25C (Ser216), LC3B, p27, p62 (SQSTM1) and cyclin B1 were purchased from Cell Signaling Technology (Danvers, MA). Monoclonal antibodies to DUSP1 (MKP-1), phospho-JNK and JNK were purchased from Santa Cruz Biotechnology (Dallas, USA). Horseradish peroxidase (HRP)-conjugated goat anti-mouse (GxMu-003-DHRPX) and HRP-conjugated goat anti-rabbit (GtxRb-003-DHRPX) secondary antibodies were obtained from ImmunoReagents Inc. (Raleigh, NC). The secondary antibody Alexafluor 488 was from Invitrogen (Carlsbad, CA). Ultra-high quality water was used to prepare all buffers and solutions. All reagents were of the purest commercial grade.

### Apple samples

*Annurca* (*Malus pumila* Miller cv. *Annurca*) apple undergoes a peculiar postharvest storage. In October, fruits are picked before the complete maturity still unripe and are subjected to the reddening on the ground for about 1 month in specially constructed boxes called “melai”. Following this phase that exalts the *Annurca* characteristics giving typicality to the product, the apple fruits (each weighing approximately 100 g) were analyzed^20^.

### Polyphenolic content and HPLC analysis

The preparation of APE was performed according to the protocol described by D’Angelo *et al*.^58^. The amounts of total pholyphenols in apple extracts were determined by Folin-Ciocalteu phenol reagent as described by Singleton *et al*.^59^, by using catechin as a reference standard and its value, expressed as milligrams of catechin equivalents (EqC)/100 g of flesh fresh weight, resulted approximately 125.2 ± 7.1 mg of catechin per 100 g of sample^58^. The determination of the polyphenolic profile of APE was performed by HPLC analysis and confirmed the results already described in the literature^21,60^.

### Cell culture

MDA-MB-231 and MCF10A cell lines were obtained from the American Type Culture Collection (ATCC, Manassas, VA). Cells were cultured in DMEM supplemented with 10% heat-inactivated FBS, 100 U/ml penicillin, 100 μg/ml streptomycin, and 1% L-glutamine.

### Cell viability

The effect of APE on cell viability was determined by the colorimetric MTT assay according to the manufacturer’s instruction. Cell viability was measured as reported by D’Angelo *et al*.^23^. Briefly, MDA-MB-231 and MCF10A cells were seeded in serum-containing media in 96-well plates at the density of 4 × 10^3^ and 5 × 10^3^ cells/well, respectively, and treated with increasing APE concentrations (from 100 to 500 μM EqC). After 24, 48 and 72 h the incubation medium was removed and MTT solution was added to a final concentration of 0.5 mg/ml. The cell viabilities were normalized to the control. The IC_50_ values were calculated by using the linear-regression analysis. All MTT assays were executed in quadruplicate.

### CellROX assay

MCF10A and MDA-MB-231 cells were plated in 24-well plates and treated with APE 500 µM EqC and APE 50, 100 and 300 µM EqC, respectively, with or without pretreatment with 5 mM NAC, for 24 h and with 100 μM menadione as a positive control for 1 h at 37 °C. 5 mM NAC was also added in menadione-treated wells. According to Mele *et al*.^61^, the cells were stained with 5 μM CellROX green reagent by adding the probe to the medium and incubating at 37 °C for 30 min. After washing with PBS, the cells were imaged on a fluorescence microscope EVOS FL Cell Imaging System (Thermo Scientific, Rockford, USA). The fluorescence intensity of CellROX assay was quantified using a BD Accuri™ C6 cytometer (BD Biosciences, San Jose, CA) and data were analyzed by FlowJo V10 software (FlowJo LLC, USA).

### Measurement of APE antioxidant effect

MCF10A cells were seeded in 96-well plates, incubated with different concentrations of APE for 24 h and then exposed to H_2_O_2_ 500 µM for 30 min. The cells were then incubated in the dark with 10 μM DCFH-DA for 1 h at 37 °C. Fluorescence was quantified in a microplate-reader (Tecan Trading AG, Switzerland) at an excitation wavelength of 485 nm and an emission wavelength of 535 nm.

### Confocal laser-scanning microscopy

MCF10A and MDA-MB-231 cells were cultured in 24-well plates containing microscope glass (12 mm) (Thermo Scientific) and treated with APE 500 and 300 µM EqC, respectively, with or without pretreatment with 5 mM NAC. For vimentin localization, cells were fixed with 4% paraformaldehyde for 20 min and permeabilized with 0.1% Triton X-100 in PBS for 10 min at room temperature. Then, the supernatant was removed and, after three time washing with PBS, the cells were incubated for 2 h at 37 °C with specific antibodies against vimentin (1:1000) followed by incubation with secondary antibodies conjugated to Alexafluor 488 (1:1000) for 1 h at room temperature. For DAPI nuclear staining the cells were incubated with 2.5 µg/ml DAPI for 7 min at room temperature in the dark. Microscopy images were performed using Zeiss LSM 700 confocal microscope equipped with a plan apochromat X63 (NA 1.4) oil immersion objective by selecting the same area as the region of interest and by setting the scan zoom to encompass only that area.

### Flow cytometry analysis of cell cycle

MDA-MB-231 cells were seeded in 6-well plates and treated for 24 h with APE 100 and 300 μM EqC with or without 1 h pretreatment with 5 mM NAC or 5 μM SP600125. Subsequently, following the procedure reported in Delle Cave *et al*.^62^ cells were harvested, washed twice with PBS and incubated with nuclei PI-staining solution for 1 h. The cell cycle distribution was measured by a BD Accuri™ C6 (Becton Dickinson, San Jose, CA).

### Flow cytometry analysis of apoptosis

Annexin-FITC was used in conjunction with the vital dye PI to distinguish apoptotic (Annexin V-FITC-positive, PI-positive) from necrotic (Annexin V-FITC-negative, PI-positive) cells. MDA-MB-231 cells were plated in 6-multiwell plates and treated for 24 h with APE 100 and 300 μM EqC, with or without pretreatment with 5 mM NAC or 5 μM SP600125 for 1 h. For the analysis of the extrinsic apoptosis pathway, cells were co-treated with 40 µM Z-IETD-FMK caspase-8 inhibitor and APE 300 µM EqC for 24 h. Then, supernatant was collected and cells were harvested by incubation with trypsin-EDTA and washed with PBS twice. Then cells were resuspended in 200 μl Binding Buffer 1X and incubated with 2 μl Annexin V-FITC and 2 μl PI (20 μg/ml). Finally, samples were analyzed by a BD Accuri™ C6.

### LysoTracker-Red staining

MDA-MB-231 cells were seeded in 6-well plates and treated with 100 and 300 μM EqC APE with or without pretreatment with 5 mM NAC or 5 μM SP600125 for 1 h. After 24, 48, and 72 h LTR was added to each well for 20 min at 37 °C at a final concentration of 0.1 μM. Cells were then washed with PBS and observed by fluorescence microscopy. Thereafter, the cells were harvested and washed twice with PBS. The fluorescence intensity was analyzed by flow cytometry and data acquired as FL-2A (linear scale) using a BD Accuri™ C6.

### Preparation of cell lysates and western blot analysis

MDA-MB-231 cells were cultured in 10-cm culture dishes and treated for 24 or 72 h with 100 and 300 μM EqC APE. Cells were processed for western blotting as described previousy^23^. Briefly, cells were harvested and lysed on ice for 30 min. The protein concentration was determined by Bradford method^63^. 20–40 μg of sample proteins were separated by SDS-PAGE and electrotransferred onto nitrocellulose membranes by Trans blot turbo (Bio-Rad Laboratories). The membrane was blocked with 5% nonfat dry milk, incubated first with specific primary antibodies at 4 °C overnight in TBST and then for 1 h with HR-conjugated secondary antibodies. Immunoblots were developed using enhanced chemiluminescence detection reagents ECL (Westar, Cyanagen, Italy), exposed to X-ray film and scanned by ImageJ software (National Institutes of Health, Bethesda, MD, USA).

### Statistical analysis

Data were expressed as mean ± standard deviation (SD) of three independent experiments with replicate samples. Data were compared with One-way ANOVA statistical test followed by Bonferroni’s t-test. *P* values less than 0.05 were considered statistically significant.

## Supplementary information

Supplementary Information
