# Supplementary material for: Annurca apple polyphenol extract selectively kills MDA-MB-231 cells through ROS generation, sustained JNK activation and cell growth and survival inhibition
Source: Sci Rep. 2019 Sep 10;9:13045. doi: 10.1038/s41598-019-49631-x (PMC6736874; doi:10.1038/s41598-019-49631-x)
Supplement: Supplementary file 1 — Supplementary Information [file 41598_2019_49631_MOESM1_ESM.pdf]

Supplementary Information

Annurca apple polyphenol extract selectively kills MDA-MB-231 cells through ROS generation, sustained JNK activation and cell growth and survival inhibition

Elisa Martino[1+], Daniela Cristina Vuoso[1+], Stefania D'Angelo[2], Luigi Mele[3], Nunzia D'Onofrio[1], Marina Porcelli[1], and Giovanna Cacciapuoti[1*]

[1] Department of Precision Medicine, University of Campania "Luigi Vanvitelli", via Luigi De Crecchio 7, 80138 Naples, Italy.

[2]Department of Motor Sciences and Wellness, "Parthenope" University, via Medina 40, 80133, Naples, Italy.

[3]Department of Experimental Medicine, University of Campania "Luigi Vanvitelli", via Luciano Armanni 5, 80138 Naples, Italy.

[+]Elisa Martino and Daniela Cristina Vuoso contributed equally to this work. [*]Correspondence and requests for materials should be addressed to Giovanna Cacciapuoti (email:giovanna.cacciapuoti@unicampania.it).

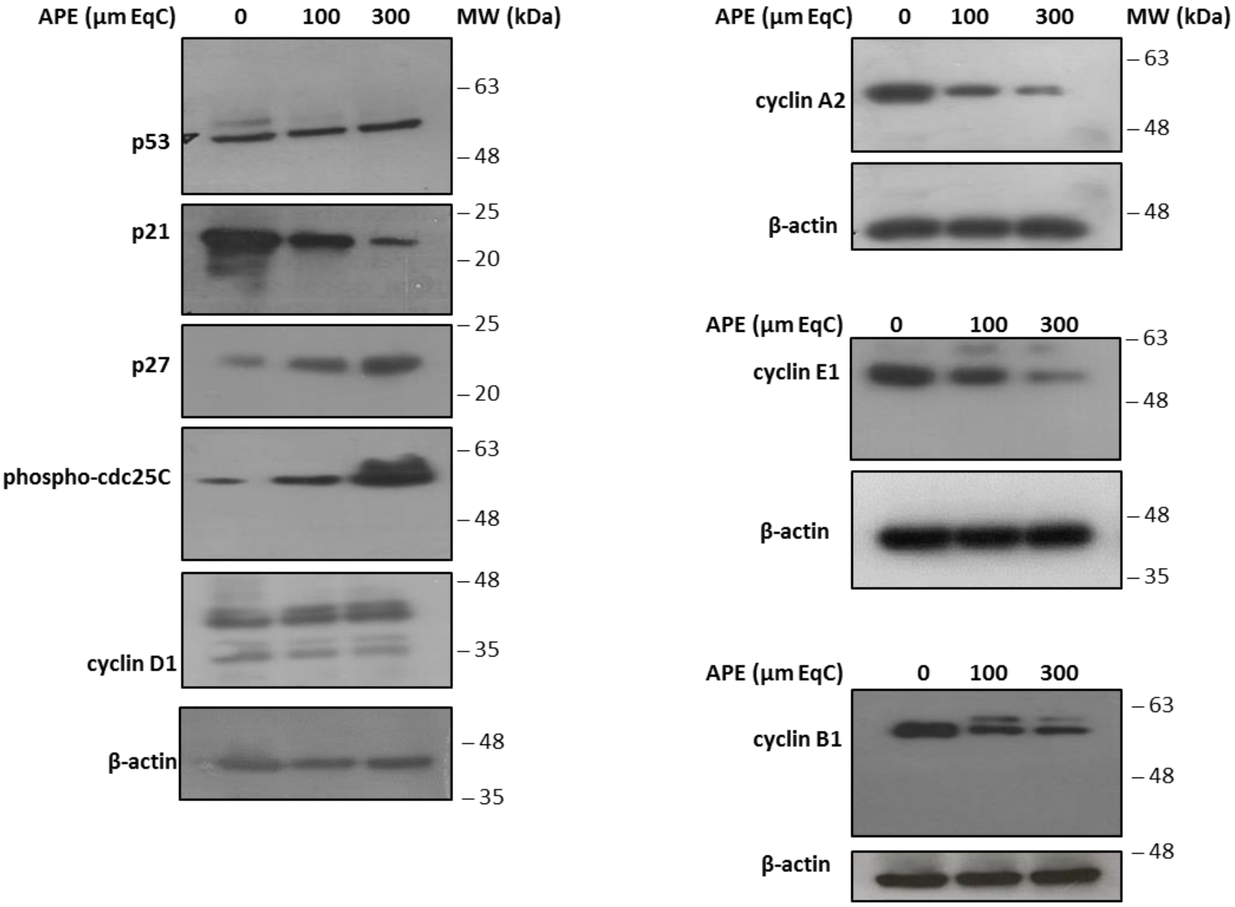

Figure S1. APE inhibits MDA-MB-231 cell growth and induces G2/M phase arrest. The cropped blots are used in the main figure (Figure 1c).

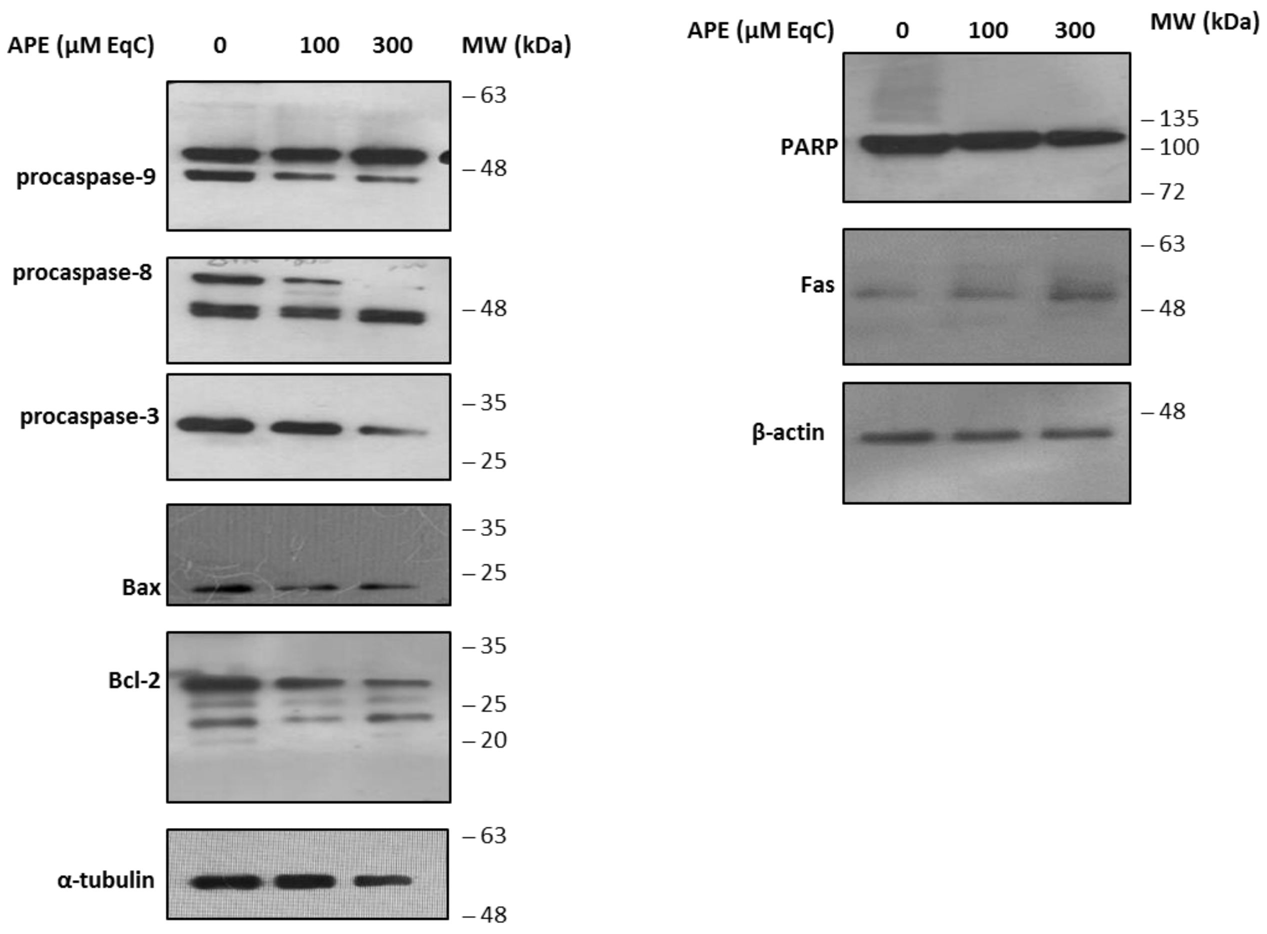

Figure S2. APE induces apoptosis in MDA-MB-231 cells. The cropped blots are used in the main figure (Figure 2b).

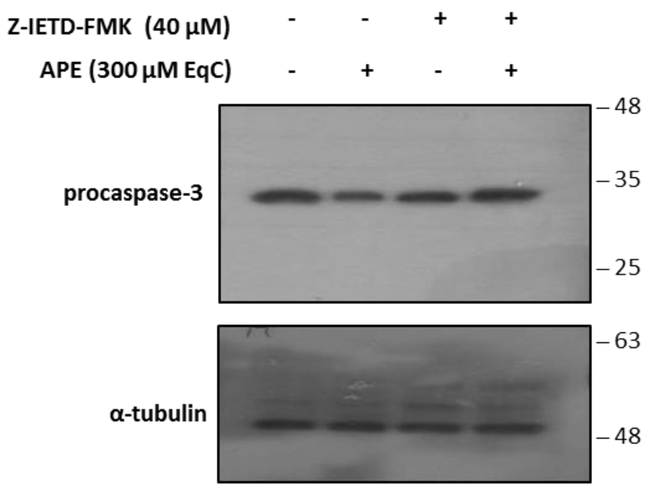

Figure S3. APE induces apoptosis in MDA-MB-231 cells. The cropped blots are used in the main figure (Figure 2d).

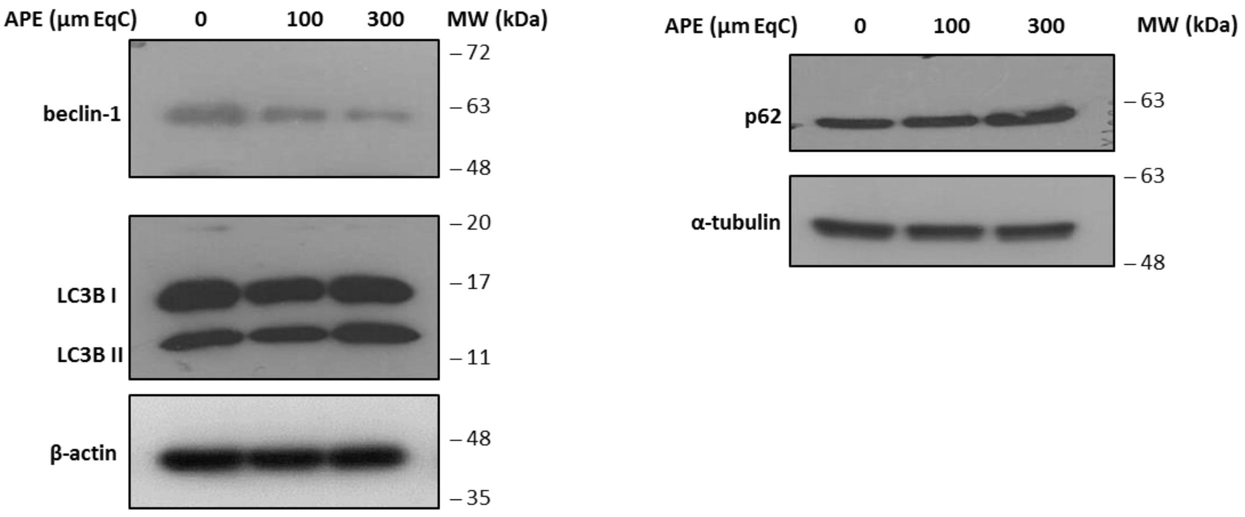

Figure S4. APE induces beclin-independent autophagy in MDA-MB-231 cells The cropped blots are used in the main figure (Figure 3c).

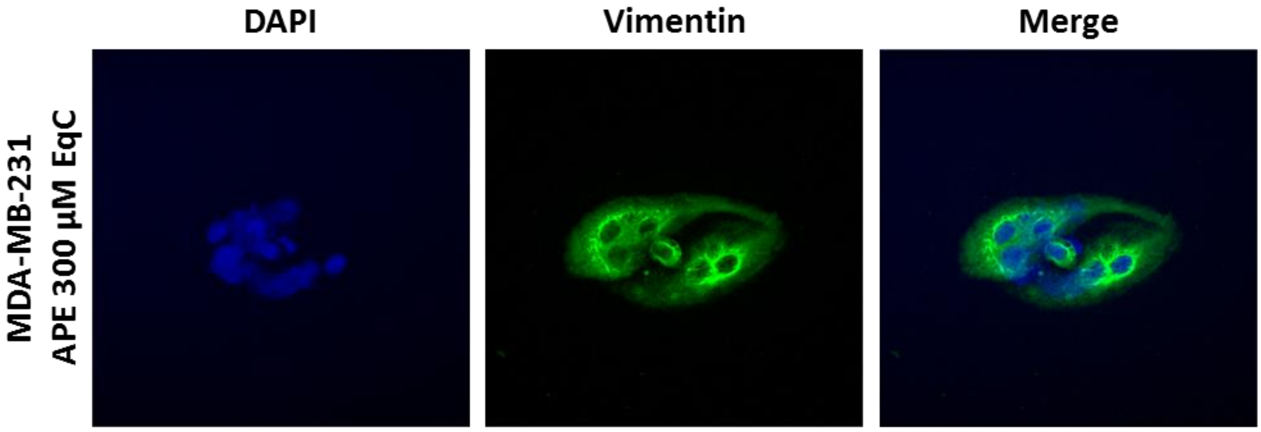

Figure S5. APE increases ROS accumulation in MDA-MB-231 cells, while it displays an antioxidant effect in MCF10A cells. Representative confocal images of the effect of APE on cell morphology of MDA-MB-231 treated with 300 µM EqC APE for 24 h. Nuclear DNA was labelled with DAPI (shown in blue), vimentin antibody (in green) was used as cytoskeleton marker. The merged image is included on the right.

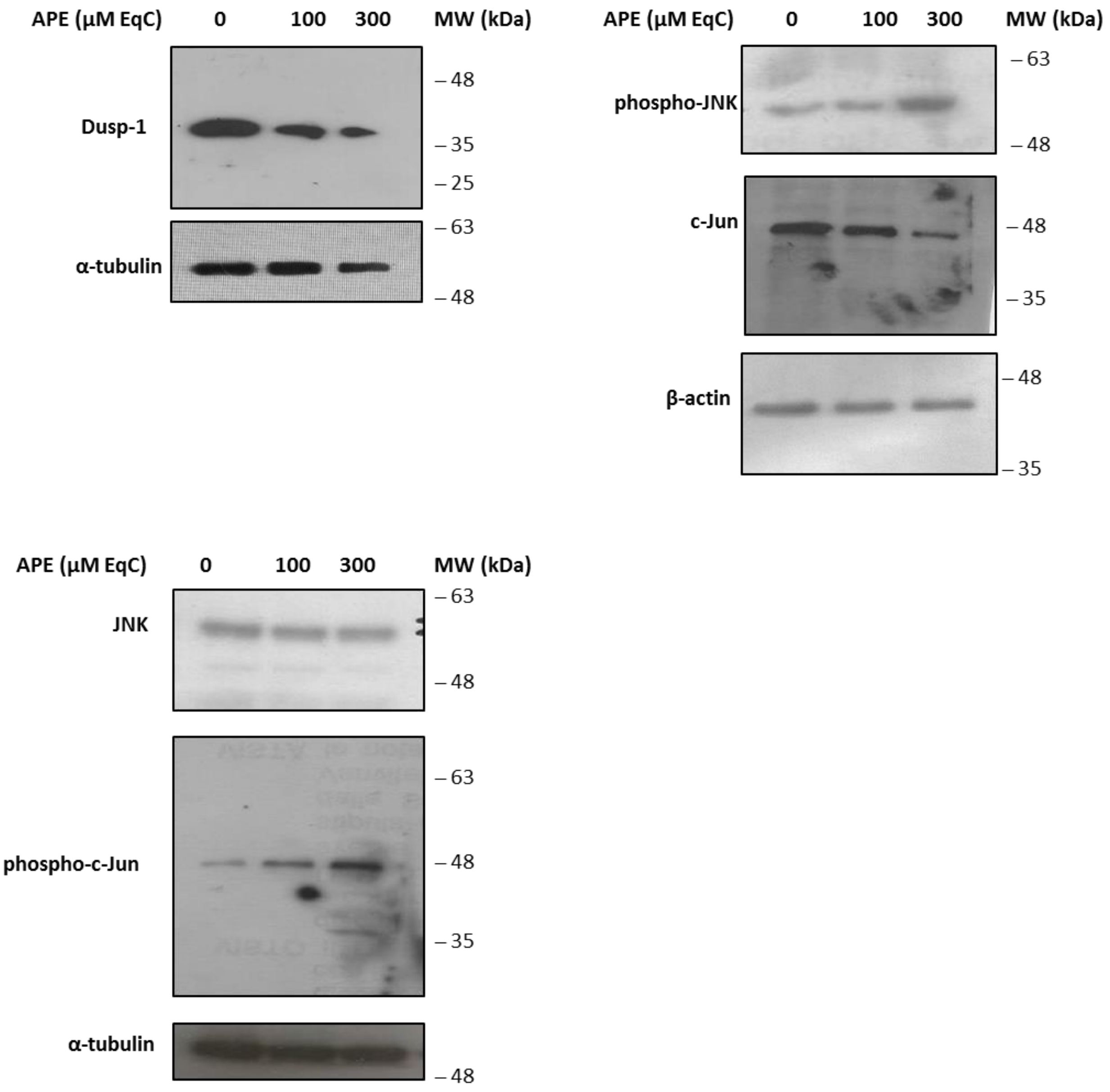

Figure S6. APE-induced ROS activate JNK/c-Jun signaling and downregulate Dusp-1 in MDA-MB-231 cells. The cropped blots are used in the main figure (Figure 5a).

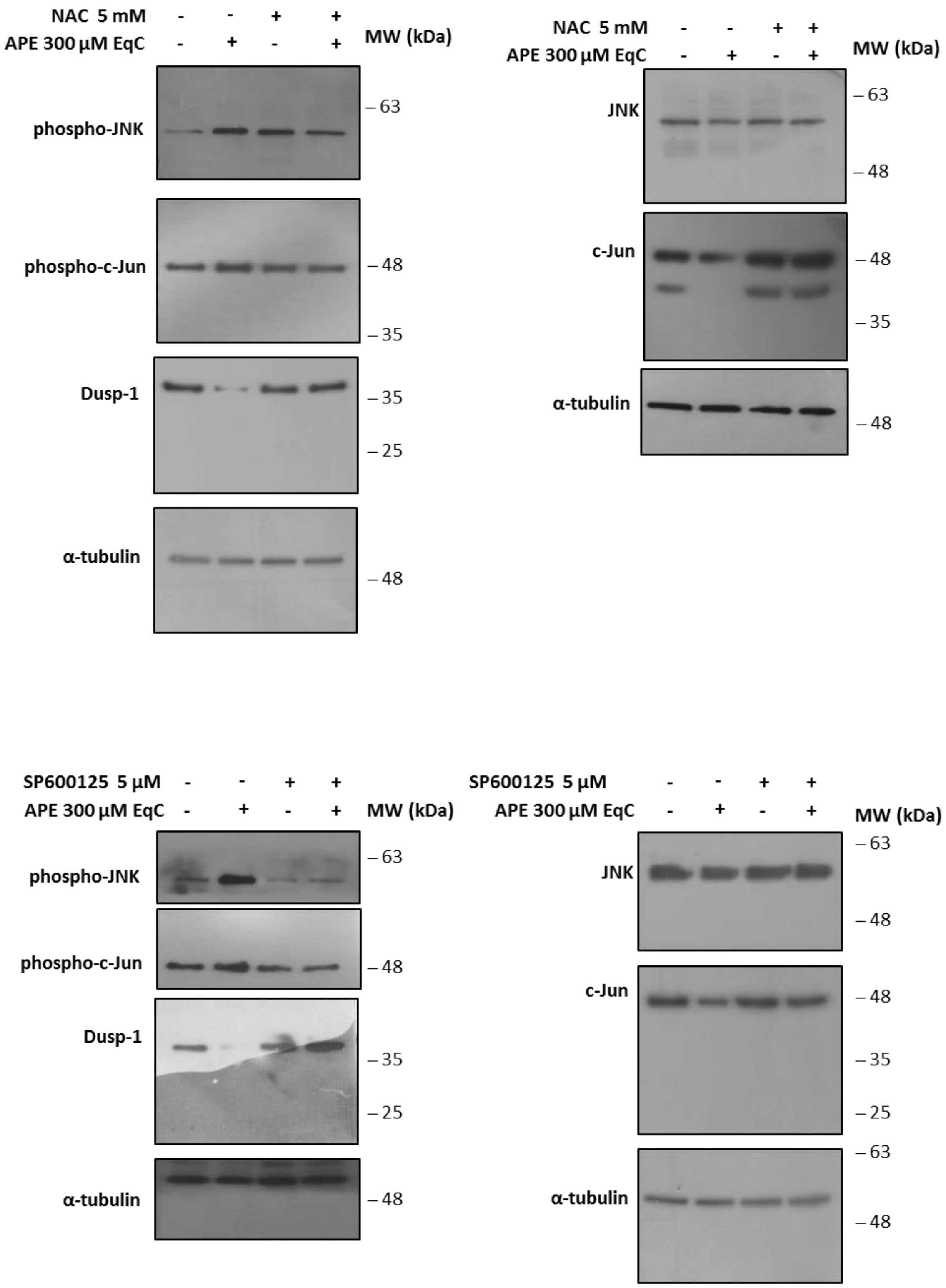

Figure S7. APE-induced ROS activate JNK/c-Jun signaling and downregulate Dusp-1 in MDA-MB-231 cells. The cropped blots are used in the main figure (Figure 5b).

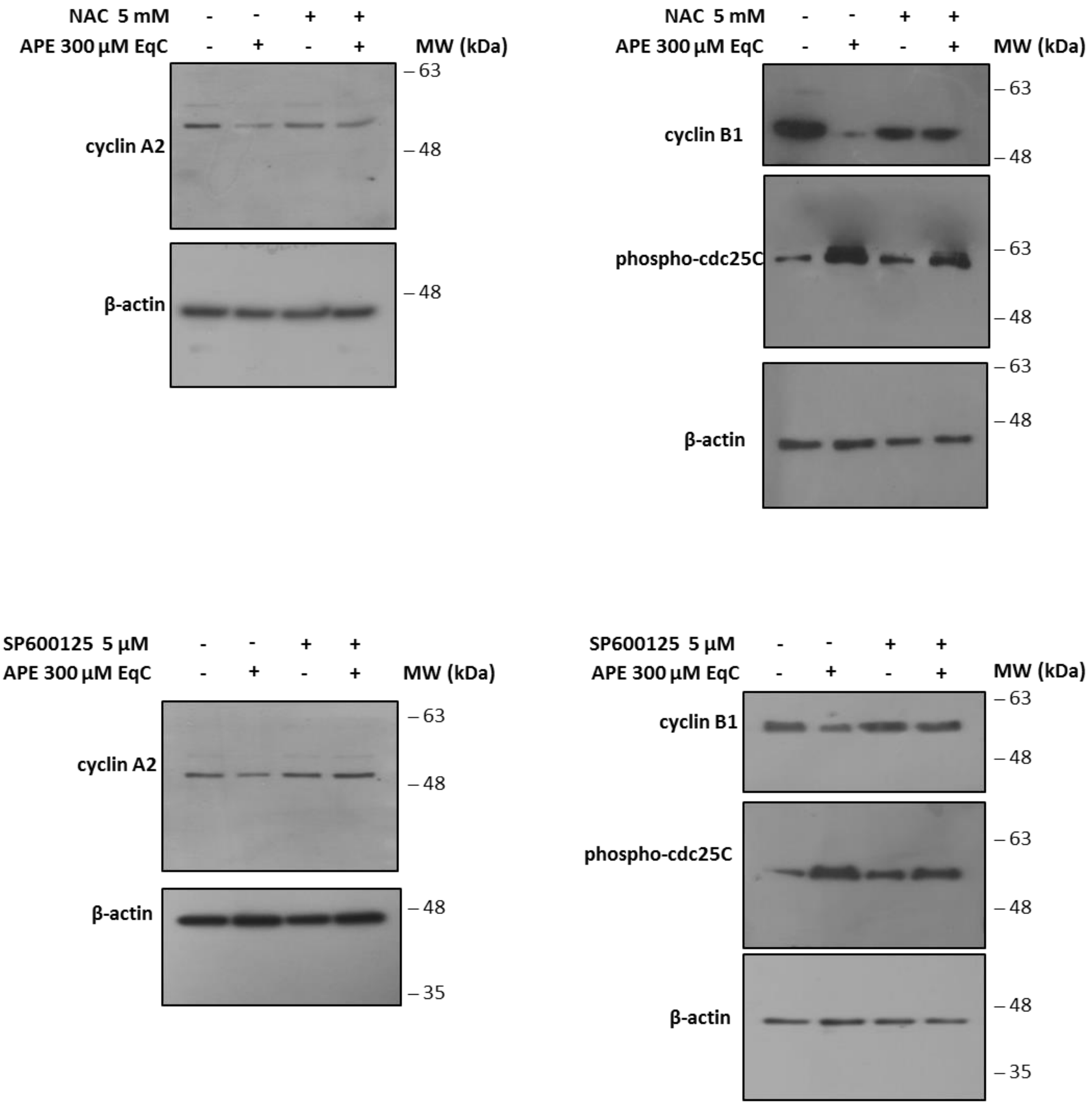

Figure S8. ROS/JNK pathway mediates APE-induced cell cycle arrest, apoptosis and autophagy in MDA-MB-231 cells. The cropped blots are used in the main figure (Figure 6a).

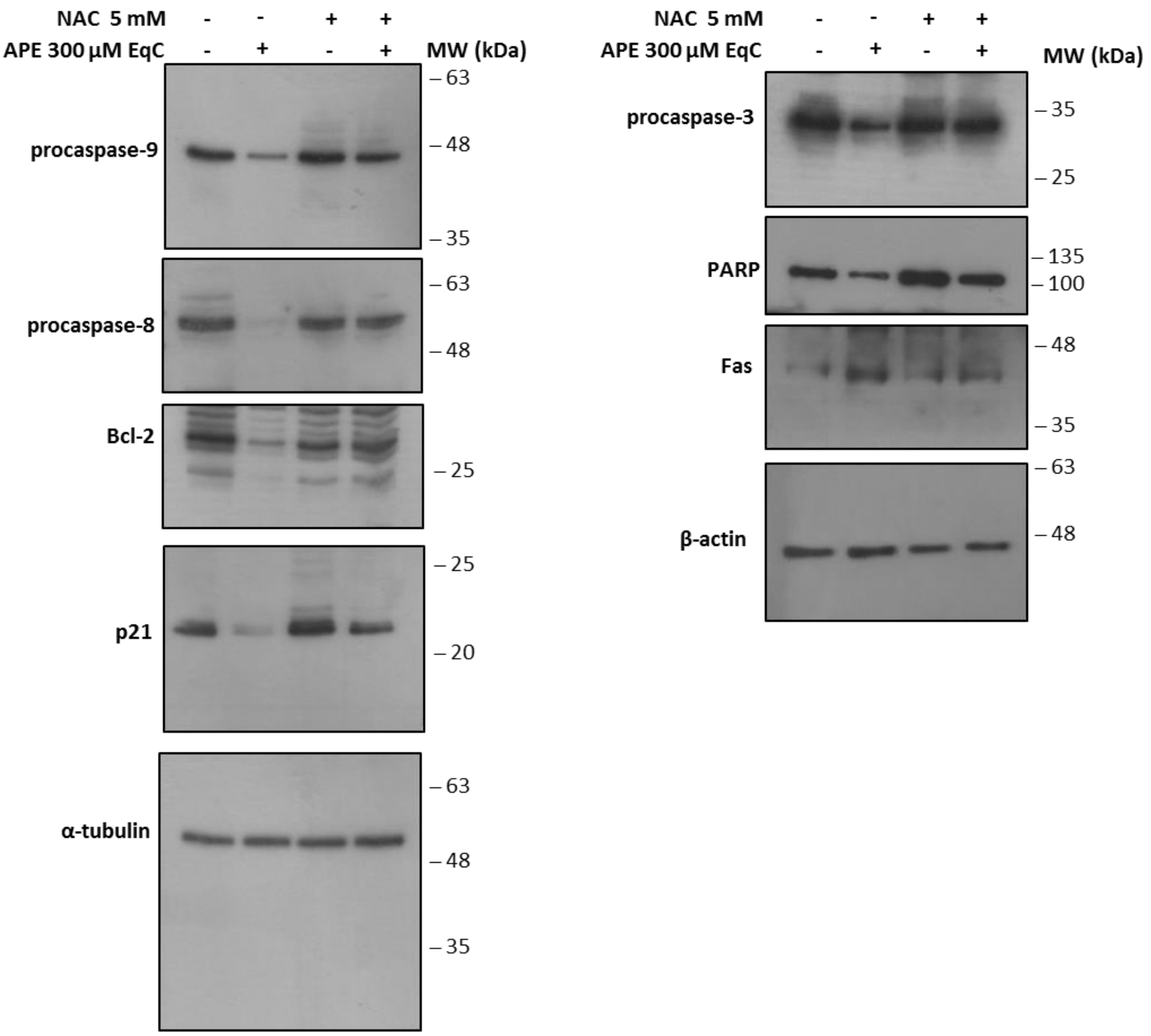

Figure S9. ROS/JNK pathway mediates APE-induced cell cycle arrest, apoptosis and autophagy in MDA-MB-231 cells. The cropped blots are used in the main figure (Figure 6b).

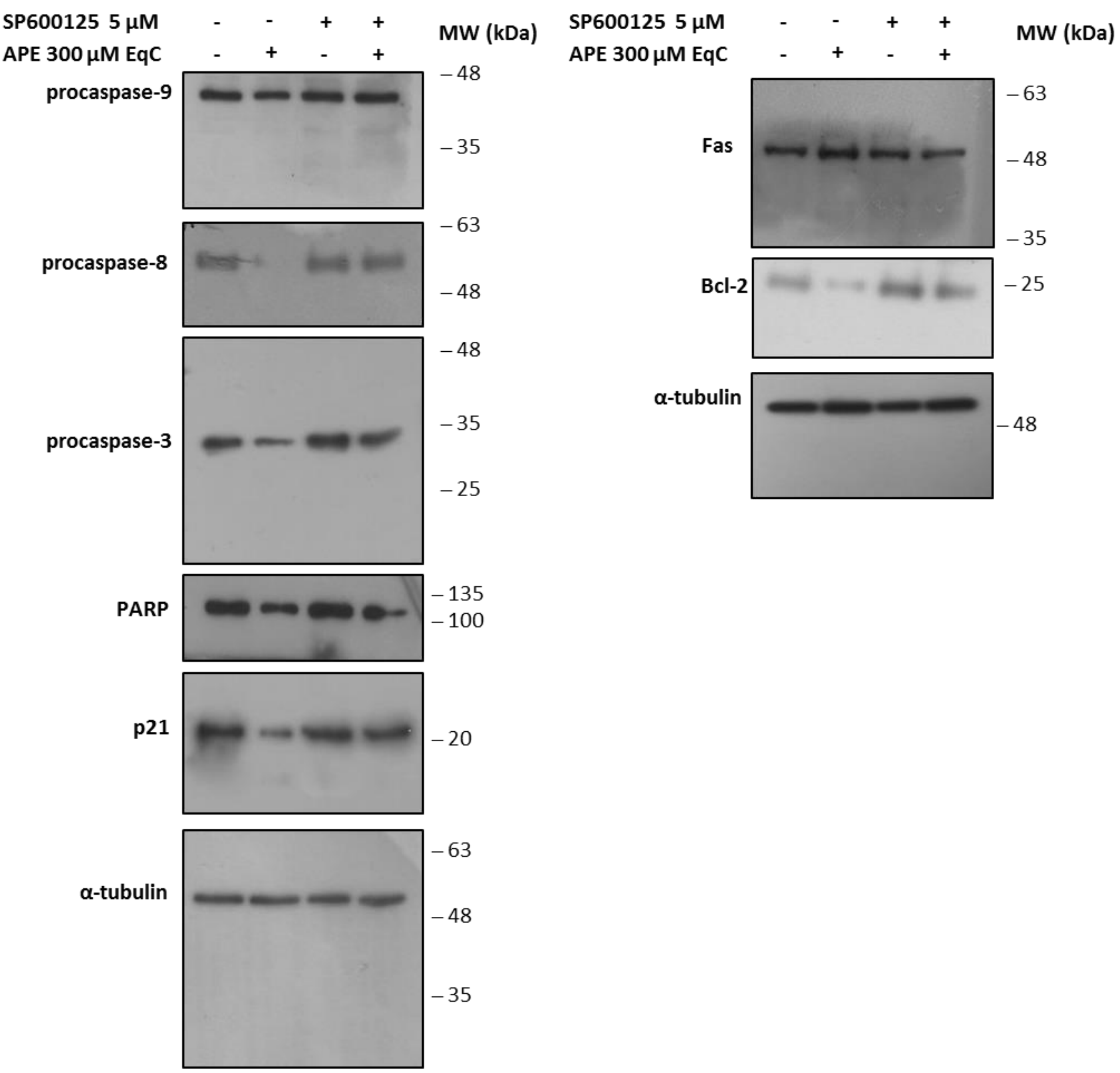

Figure S10. ROS/JNK pathway mediates APE-induced cell cycle arrest, apoptosis and autophagy in MDA-MB-231 cells. The cropped blots are used in the main figure (Figure 6b).

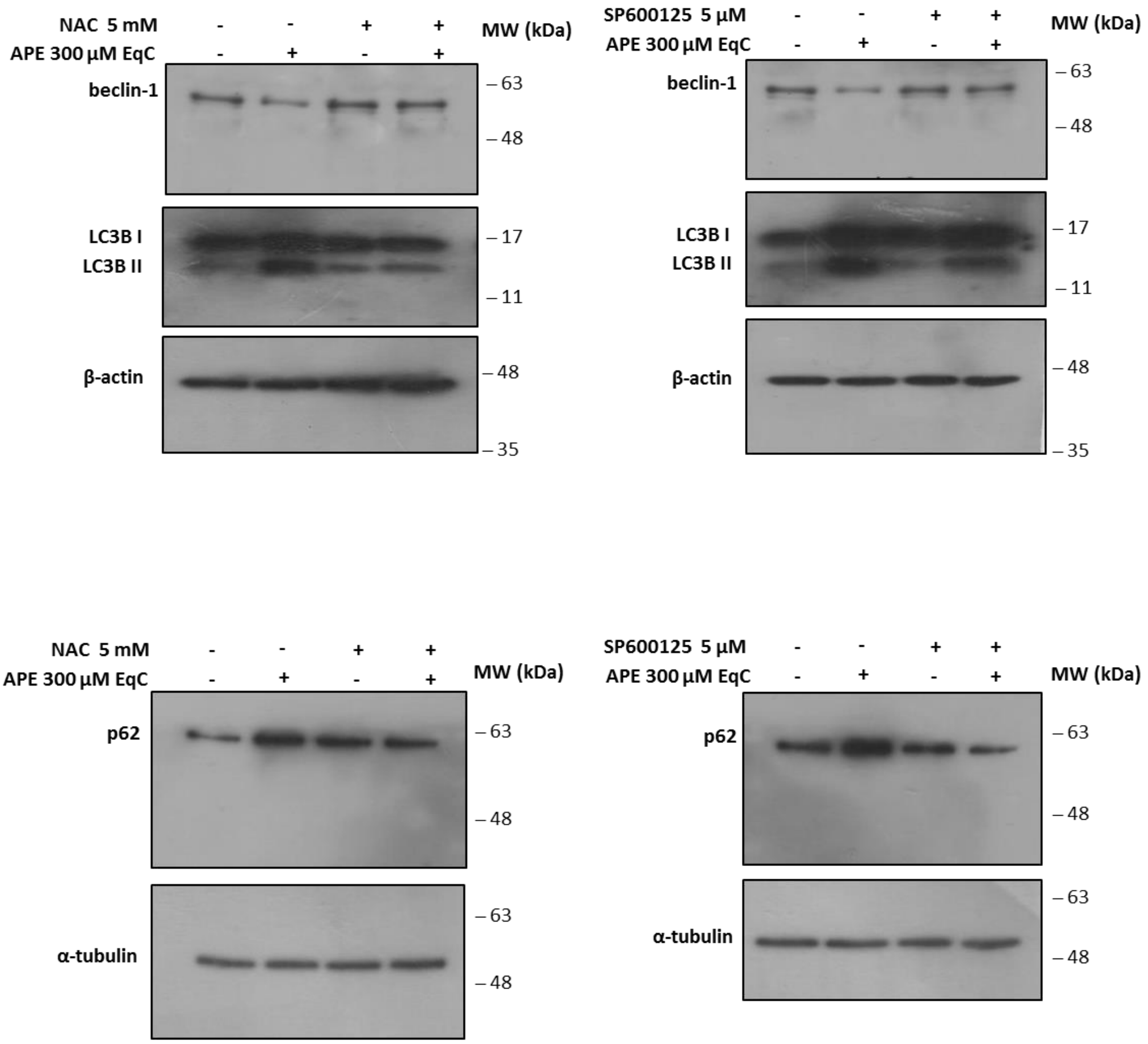

Figure S11. ROS/JNK pathway mediates APE-induced cell cycle arrest, apoptosis and autophagy in MDA-MB-231 cells. The cropped blots are used in the main figure (Figure 6c).

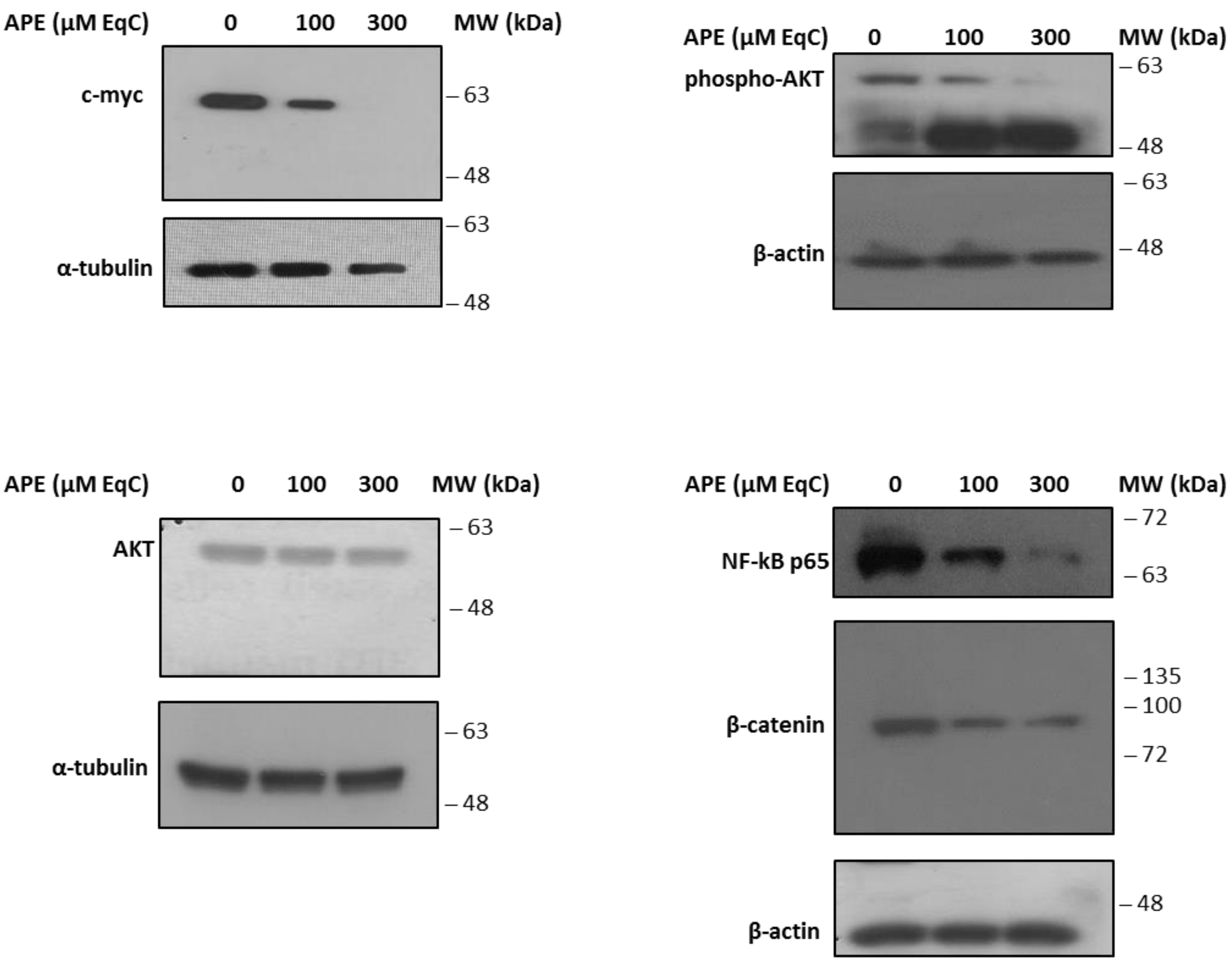

Figure S12. APE downregulates the main cell growth and survival pathways. The cropped blots are used in the main figure (Figure 7a).

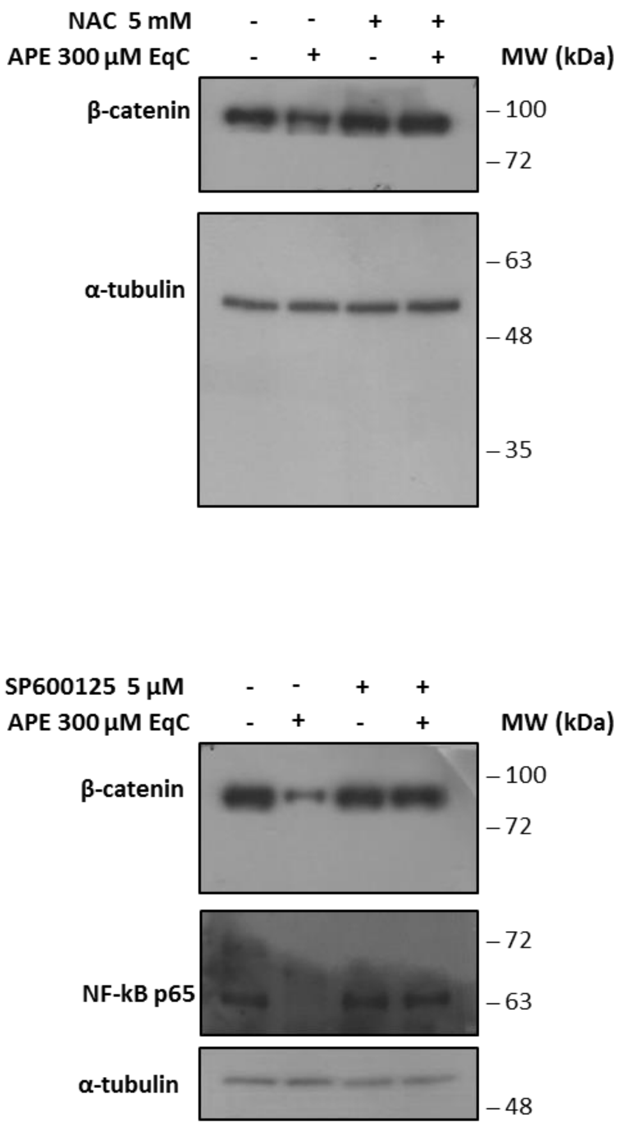

Figure S13. APE downregulates the main cell growth and survival pathways. The cropped blots are used in the main figure (Figure 7b).
